# Genome-wide analysis of the *HSP101/CLPB* gene family for heat tolerance in hexaploid wheat

**DOI:** 10.1038/s41598-020-60673-4

**Published:** 2020-03-03

**Authors:** Eva Erdayani, Ragupathi Nagarajan, Nathan P. Grant, Kulvinder S. Gill

**Affiliations:** 1 Department of Crop and Soil Sciences, Washington State University, Pullman, WA. USA; 2 Present Address: Research Center for Biotechnology, Indonesian Institute of Sciences, Cibinong, Jawa Barat Indonesia

**Keywords:** Gene expression profiling, Abiotic

## Abstract

Heat Shock Protein 101 (HSP101), the homolog of *Caseinolytic Protease* B (CLPB) proteins, has functional conservation across species to play roles in heat acclimation and plant development. In wheat, several *TaHSP101/CLPB* genes were identified, but have not been comprehensively characterized. Given the complexity of a polyploid genome with its phenomena of homoeologous expression bias, detailed analysis on the whole *TaCLPB* family members is important to understand the genetic basis of heat tolerance in hexaploid wheat. In this study, a genome-wide analysis revealed thirteen members of *TaCLPB* gene family and their expression patterns in various tissues, developmental stages, and stress conditions. Detailed characterization of *TaCLPB* gene and protein structures suggested potential variations of the sub-cellular localization and their functional regulations. We revealed homoeologous specific variations among *TaCLPB* gene copies that have not been reported earlier. A study of the Chromosome 1 *TaCLPB* in four wheat genotypes demonstrated unique patterns of the homoeologous gene expression under moderate and extreme heat treatments. The results give insight into the strategies to improve heat tolerance by targeting one or some of the *TaCLPB* genes in wheat.

## Introduction

Increasing global temperature is a serious concern in agriculture as it affects crop productivity and food production. Heat stress often causes irreversible damages to plant physiological process and development. Wheat, one of the major cereal crops grown worldwide, is highly sensitive to heat stress. Frequent occurrence of days with super optimal temperatures during a wheat growing season directly affects yield^1,2^. At cellular level, high temperature damages membranes of different sub-cellular compartments and degrades proteins^3,4^. Kinetic activities of enzymes involved in photosynthesis, respiration, cell division, and many other vital processes are also affected by heat stress^5–8^.

*Caseinolytic Protease B* (CLPB) proteins play important roles in organisms, especially in stress response and at different developmental stages. These proteins are high molecular weight chaperones that are part of the Heat Shock Protein 100 (HSP100) family^9^. The initial study on one of the CLPB members in yeast discovered the role of Heat Shock Protein 104 (HSP104) in heat acclimation^10^. A process that involves protein disaggregation by HSP104 was later observed as the mechanism of heat tolerance in yeast^11^. The orthologous proteins were also identified in other organisms with similar functional characteristics to yeast HSP104. Bacterial CLPB^12,13^ and plant HSP101 or CLPB1^14–16^ were characterized as the functional orthologs of yeast HSP104. In plants, beside HSP101 that is localized in the cytoplasm, the homologs of CLPB were also identified within the plastid and mitochondria^17,18^. Plastid localized CLPB in *Arabidopsis thaliana* (*Arabidopsis*) was known to play a role in plastid development and plant survival^19,20^, while the ortholog in tomato was known to be important for heat acclimation^21^.

Detailed structural features of CLPB protein were initially characterized by Lee *et al*. (2003) in the model bacterium *Thermus thermophilus*. The protein was described as a two-tiered ring of hexamers connected with coiled-coil linkers. The monomers are comprised of five conserved domains: the N-terminal domain; the D1-large domain (Nucleotide Binding Domain 1/NBD1); the D1-small domain including linker region; the D2-large domain (Nucleotide Binding Domain 2/NBD2); and the D2-small domain. CLPB was also classified as the member of the AAA+ (ATPases associated with diverse cellular activities) superfamily of ATPases^22^. The C-terminal domain of the NBD2 was predicted to be critical for CLPB oligomerization while the interaction of ATP with the NBD1 stabilizes the CLPB oligomer^23,24^. The N-terminal domain was not found to be essential for oligomerization and disaggregation activity, but was important for CLPB binding with specific substrates such as casein^25^.

To be active, CLPB requires energy from ATP hydrolysis that is triggered by the interaction of coiled-coil linkers of the middle domain with the DnaK-DnaJ complex^26^. During protein disaggregation, DnaK-DnaJ (HSP70-HSP40) exposes the peptide segment of damaged proteins to the central pore of the CLPB hexamer which will progressively pull and unfold it^27^. The long coiled-coil structure of the linker region has been known as a characteristic feature that distinguishes CLPB from the other AAA+ or the HSP100 family members^28^. In *Thermus thermophillus*, the CLPB linker forms a two-bladed propeller with two motifs that is similar to that of leucine zippers in eukaryotic transcription factors^29,30^. It was predicted that HSP70 triggers the active state of CLPB by its interaction with the CLPB linker region which acts as a molecular toggle^31,32^. A species-specific characteristic is also possibly present in the middle domain structure as shown by a specific interaction of the linker region from *E. coli* CLPB and the yeast HSP104 with DnaK and HSP70, respectively^33^.

In hexaploid wheat *Triticum aestivum*, several studies revealed the presence of *HSP101* gene copies and their expression under high temperature or other types of stress treatments. The first wheat ortholog of HSP101 was identified as a 102 kDa Ω-binding protein that can complement the thermotolerance defect in yeast *hsp104*^34^. The protein was also shown to act as a translational regulator of Ferredoxin-1 (Fed-1)^34–36^. The other two genes of wheat *HSP101* were cloned later and named as *TaHSP101B* and *TaHSP101C*, while the first HSP101 was renamed as *TaHSP101A*^37^. An *in-silico* study of the *Caseinolytic Protease Class* I family has predicted five members of the CLPB family in wheat: three of them are cytoplasmic copies and one copy each of the other two is targeted to the chloroplast and mitochondria^38^. These genes were shown to be differentially expressed at different tissues and stress conditions^37,38^. Cytoplasmic CLPBs were up-regulated in leaves under heat, salt and oxidative stress^38^. The increased expression of wheat CLPBs was also observed under drought stress, but not observed under chilling and wounding treatments^37,38^.

The wheat genome has its own complexity due to polypoidy. Genus *Triticum*, with 7 as the monoploid number of chromosomes [1x = 7], consists of diploid [2n = 2x = 14], tetraploid [2n = 4x = 28] and hexaploid wheat [2n = 6x = 42] species^39^. During the evolution of hexaploid wheat, the A genome came from *Triticum urartu* [AA]^40^, which is similar to *Triticum monococcum;* however, the B genome donor, *Aegilops speltoides*, is still controversial^41,42^. The A and the B genomes then combined to form *Triticum turgidum* [AABB]^43^ and the Allohexaploid *Triticum aestivum* [AABBDD] arose from a spontaneous hybridization of *T. turgidum* with the donor of the D genome *Aegilops tauschii*^44^. The term *homoeolog* or *homeolog* refers to genes or chromosomes that are inherited from different progenitors through interspecific hybridization, resulted in allopolyploidization^39,45^. It is distinguishable with *homolog* which refers to the genes or genomes that share similarities which are inherited from common ancestors^45^.

Differential expression of homoeologous genes are common phenomena in polyploids. Reconciliation of genomes gave consequences to the anomaly of gene expression patterns and phenotypes by the presence of changes at the genetic and epigenetic levels^46–48^. Homoeolog expression bias is unequal expression among the homoeologs at different tissues or developmental stages; or as anomaly of their expression level relative to their diploid progenitors^47,49^. Subgenomic preferences have been reported in octoploid strawberry with a single subgenome exhibited significant dominance in gene expression and control of certain metabolomic and disease resistance traits^50^. Contribution of homoeolog expression dominance in facilitating selection of glucosinolate and lipid metabolism genes was also reported in the vegetable-use and oil-use sub-varieties of *Brassica juncea*^51^. In cotton and wheat, alterations of expression patterns among homoeologs under variation in stress conditions, tissues, and developmental stages have also been documented^52–57^. Given the consequences of unequal expression to the natural gene selections in polyploids, it is important to understand the genetic basis of valuable traits with respect to the homology and homoeology perspectives for the success of selective breeding programs.

Previous studies have identified *HSP101*/*CLPB* copies in wheat without a clear map of the whole gene family in the genome of this polyploid species (Wells *et al*. 1998; Campbell *et al*. 2001; Muthusamy *et al*. 2016). In tetraploid wheat *Triticum turgidum* subsp. *durum*, four copies of *TdHSP101* were cloned and physically mapped on the two homoeologous chromosomes of groups 1 and 3^58^. Orthologs of the two genes were also placed on the corresponding chromosomes of the A genome progenitor, *Triticum monococcum*^58^. The corresponding gene copies are not known in the hexaploid wheat. Since more than one sequence were reported as the putative HSP101/CLPB homologs in wheat, there has been confusion on how many genes exactly present in the genome and functional, which copies are mainly playing role and potential to be targeted for crop improvement. Besides, lack of thorough observation on the entire gene family will potentially introduce bias in gene expression analyses due to high similarities among homologous or homoeologous genes. The bias might lead to inaccurate predictions about gene responses and functions. Specificity in gene targeting even more crucial if genome editing is the choice for genetic modification as currently has become a trend in today’s methods^59,60^. Hence, detailed analysis on all the HSP101/CLPB family members is required.

In this study, we identified all the members of *HSP101*/*CLPB* gene family in hexaploid wheat and located their position on wheat chromosomes. The sequences were characterized based on their predicted protein structures as compared to the known HSP101/CLPB sequences from two model species, rice and *Arabidopsis*. Unique conserved domains and motifs were analyzed throughout the linker regions to study variation of the proteins at the functional level. Gene expression patterns were characterized *in silico* and in real time PCR with respect to plant developmental stages and stress treatments. *TaCLPBs* of the group 1 chromosomes, of which a member was shown to complemet the *hsp104*^34^, were found to be more responsive to drought and heat stress. We specifically cloned and studied this group members for their homoeologous expression patterns in four wheat genotypes under moderate and extreme high temperatures.

## Materials and Methods

### Identification of *CLPB* gene copies and their mapping to the wheat genome

Using a tblastx tool^61,62^, the rice sequences (*Os05g0519700*, *Os03g0426900*, *Os02g0181900*) were used as references and queries to retrieve the orthologs from the wheat sequence databases in NCBI (https://www.ncbi.nlm.nih.gov), Swissprot/Uniprot (http://www.uniprot.org), and EnsemblPlants (http://plants.ensembl.org/Triticum_aestivum). The retrieved sequences were then confirmed as orthologs of CLPB following the criteria developed by Dhaliwal *et al*.^63^. Briefly, orthologous sequences have to meet four criteria: the highest level of sequence identities and query coverage, the presence of domains and motifs of CLPB at the protein level, the relative size and distance among domains and motifs to be similar to the query, and that orthologs must retrieve the reference sequences at the first place when the basic local alignment (BLAST) against nucleotide or protein databases are performed. Ensemble Plants database was used to confirm the ancestral relationship of the putative sequences with the orthologous genes from other species.

The *TaCLPB*s gene family were mapped on wheat chromosomes using the BLAST (tblastn) tool in the Wheat Chromosome Survey Sequence (https://wheat-urgi.versailles.inra.fr/Seq-Repository) database generated by the *International Wheat Genome Sequencing Consortium* (https://www.wheatgenome.org/About). In this early wheat database, individual chromosome arms were derived and sequenced from double ditelosomic stocks of the hexaploid wheat cultivar Chinese Spring^62^. Partial sequences retrieved during the analysis were recovered through a sequence search in: (1) the NCBI EST database of bread wheat (https://www.ncbi.nlm.nih.gov/dbEST/); (2) the draft assembly of gene rich regions of Chinese Spring wheat in the Cereal Database (http://www.cerealsdb.uk.net/cerealgenomics/CerealsDB); the genome database of wheat progenitors, including *Triticum urartu*, *Aegilops speltoides*, *Aegilops tauschii*, and *Triticum turgidum* subsp. durum (https://urgi.versailles.inra.fr/download/iwgsc/TGAC_WGS_assemblies_of_ other_wheat_species/). Full length sequence contigs were synthesized by a manual assembly of the partial sequences through their overlapping regions using the DNA alignment tool in Clustal Omega tool (https://www.ebi.ac.uk/Tools/msa/clustalo/). Recent updates with the release of IWGSC RefSeq assembly v1.1 (https://urgi.versailles.inra.fr/download/iwgsc/IWGSC_RefSeq_Assemblies/v1.0/), were incorporated later in the analysis and mostly confirmed the manual assembly and annotation in the previous analysis.

### Analysis of CLPB genes and proteins

*TaCLPB* putative genes were aligned with the known cDNA/EST sequences by using a DNA alignment tool in Clustal Omega to manually identify the exon and intron junctions. Predicted CDS sequences were translated into protein sequences using the EMBOSS Transeq translation tool (https://www.ebi.ac.uk/Tools/st/emboss_transeq/). The translated sequences were then used to analyze homology among *TaCLPB* proteins under multiple sequence alignment using a protein alignment tool in Clustal Omega. Protein conserved domains were identified using the NCBI’s CD-Search tool (https://www.ncbi.nlm.nih.gov/Structure/cdd/wrpsb.cgi) with SMART and Pfam databases as references. To predict subcellular localization of CLPB proteins, the ChloroP 1.1 Server^64^ and the TargetP 1.1 Server (http://www.cbs.dtu.dk/services)^65^ were used to identify signatures of signal peptides. Default parameters were used for all the analyses. Phylogenetic analysis was done using Maximum Likelihood method by RAxML v.8.2.12 on the CIPRES Science Gateway with the GTR + Γ model of evolution^66^. Bootstrap analyses of 1000 replicates were used as the support for the optimum maximum likelihood tree. The phylogenetic tree was visualized using Dendroscope v.3.5.9^67^.

The 3D structure of CLPB proteins was predicted using the I-TASSER software^68^. The predicted models with the highest C-score values were used to identify the ligand binding residues using the COACH program^69^. Comparisons among protein models were performed in the PDBeFold (http://pdbe.org/fold/). CLPB protein sequences of OsHSP101, OsCLPB-C, OsCLPB-M (UniProt ID: Q6F2Y7, Q75GT3, Q0E3C8); AtCLPB-1, AtCLPB-3, AtCLPB-4 (UniProt ID: P42730, Q9LF37, Q8VYJ7); and TCLPB (UniProt ID: TTHA1487) were included in the protein analysis as references to represent the models of functionally characterized CLPBs from rice, *Arabidopsis*, and *Thermus thermophilus*.

### *In silico* RNA-seq expression analysis

The manually annotated and mapped *TaCLPB* sequences were compared to latest version of the gene models (Wheat RefSeq v1.1) in EnsemblPlants. The expVIP tool was used to analyze the expression of *TaCLPB* copies *in silico* by retrieving the RNA-seq expression data of *TaCLPB* transcripts in polyploid wheat^56^. Two datasets were selected for the analysis: 1) the wheat development time course (ENA: ERP004714) and 2) the drought and heat stress (SRA: SRP045409). The expression values were visualized as the unit of transcript per kilobase exons per million reads (TPM). Differential expression analysis was done on the transcript raw-count data by the EdgeR package version 3.24.2 in the R program^70^.

### Real-time expression analyses

Real-time gene expression for five chromosomal group of *TaCLPB* members was analyzed in PBW343 variety under normal temperature (22 °C) at three developmental stages: seedling stage, anthesis stage, and grain filling stage (7DAA). In three biological replicates, four types of tissue collected were seedling leaves, mature leaves (second leaves), flag leaves, and spikes. Five primer pairs were designed as common primers to amplify *TaCLPB* members of each chromosomal group with 150–200 bp expected amplicon size.

The expression study of the homoeologous *TaCLPB* members of the chromosome 1 was done under control and heat treatments on four wheat genotypes: Chinese Spring, Red Fife, Giza 168, and PBW 343. Chinese Spring was chosen as the reference accession for wheat as its genome was sequenced. The other three genotypes are varieties originated from three different regions that are considered to pose unique temperature or climate regime. Giza 168 is a variety from Egypt, Red Fife is originated from Canada, and PBW 343 from India. Leaf samples were collected from three biological replicates of 12-day old seedlings following the treatments of (1) 2 h at 37 °C; (2) 4 h at 37 °C; and (3) 2 h at 37 °C plus 4 h at 42 °C. Homoeologous specific primers were designed to amplify around 200–300 bp amplicons and the specificity of each was tested using Chinese Spring Nullisomic-tetrasomic lines for the group I chromosomes by PCR amplification. All primers were listed in the Supplement 1.

Total RNA was isolated using a modified Hot Phenol Extraction method^71^ and the cDNA was synthesized using M-MLV Reverse Transcriptase enzyme kit (Promega, WI, USA). Relative transcript abundance was measured by Real-time qPCR using the SYBR Green I detection system from Kapa Biosystem for Roche LightCycler 480. PCR mixtures were composed of 50x dilution of cDNA samples (synthesized from 1 µg RNA), 0.2 pmol/µl primers, 1.2x Kapa Sybr Fast LC480 (Kapa Biosystems, USA). As the amplicons were expected to have high GC contents, 2.5% DMSO was added into the reaction. The cycling conditions were 95 °C/3 min pre-incubation; 32 cycles of 95 °C/10 sec denaturation, 62 °C/20 sec annealing, 72 °C/1 sec extension. Data analyses were done using the LinReg PCR program^72^. The expression levels are shown as the means of normalized ratios of the target gene to the actin gene expression along with the standard deviation of three biological replicates^73^. The fold change values represent the ratio of the target gene expression as compared to the control with the error bars showing relative standard deviations (rsd) of three biological replicates^73^. Statistical analysis of the expression data was done by the analysis of variance (anova) for multiway-treatment structure, followed by a post-hoc multiple comparison using Tukey’s test in the R program.

### Cloning of *TaCLPB* homoeologous copies from the group 1 chromosome

Based on our sequence analysis, the *TaCLPBs* of the chromosome 1 are the functional orthologs of HSP101. *TaCLPB* in the chromosome 1A has been shown to complement yeast hsp104 mutant^34^. It became of interest to identify all the homoeologs in this group by sequence cloning and characterize the homoeologous expression patterns. The putative full-length sequences of the chromosome 1 *TaCLPB* members were used as references for designing homoeologous specific primers (Supplement 1). The PCR reactions were performed on the Chinese Spring genomic DNA and cDNA templates. Amplicons with the expected size were cloned using the Gateway cloning system (Invitrogen, USA) and sequenced (at least three colonies per clone).

## Results and Discussions

### *TaCLPB* gene family members and their chromosomal locations in polyploid wheat

The rice CLPB proteins that have been annotated and functionally characterized are: (1) cytoplasm targeted CLPB (CLPB-c) that is also known as rice HEAT SHOCK PROTEIN 101 (OsHSP101); (2) plastid targeted CLASS I CLP ATPASE B-C (OsCLPB-C); and (3) mitochondria targeted CLASS I CLP ATPASE B-M (OsCLPB-M). Using these three proteins as references for sequence search and annotation, 13 wheat sequences were identified as the members of the wheat CLPB family; seven of which were predicted to be targeted to the cytoplasm and physically mapped to the long arm of chromosomes 1A, 1B, 1D, 3A, 3B, 3D, and 4B (1AL, 1BL, 1DL, 3AL, 3BL, 3DL, 4BL), respectively; three putative plastid targeted sequences are mapped to the long arms of the chromosome 5A, 4B, and 4D (5AL, 4BL, 4DL); three putative mitochondria targeted sequences mapped to the short arms of the chromosome 6A, 6B, and 6D (6AS, 6BS, 6DS), respectively. The alignment of *TaCLPB* sequences with their orthologs in rice are given in the Supplements 2–4. Table 1 listed the information of *TaCLPB* sequences by the genome analysis. The sequence names are symbols to differentiate the sequences based on the chromosomal location (with the addition of “1” in “*TaCLPB-4B1*” for an extra copy at the chromosome 4B). Information about the synteny of these sequences with the orthologs from other species was provided in the Supplement 5.

**Table 1:** *TaCLPB* sequences with respect to their corresponding ortholog in rice, chromosomal mapping and subcellular target locations.

No.	*TaCLPB*	Map	EnsemblPlants ID (RefSeq. 1.1 gene model)	Gene length	Aa #	Predicted sub-cellular target	Transit peptide length
1	*TaCLPB-1A*	1AL	TraesCS1A02G340100	2757	918	cytoplasm	NA
2	*TaCLPB-1B*	1BL	TraesCS1B02G352400	2754	917	cytoplasm	NA
3	*TaCLPB-1D*	1DL	TraesCS1D02G342100	2757	918	cytoplasm	NA
4	*TaCLPB-3A*	3AL	TraesCS3A02G274400	2742	913	cytoplasm	NA
5	*TaCLPB-3B*	3BL	TraesCS3B02G308100	2742	913	cytoplasm	NA
6	*TaCLPB-3D*	3DL	TraesCS3D02G273600	2742	913	cytoplasm	NA
7	*TaCLPB-4B1*	4BL	TraesCS4B02G393100	2712	903	cytoplasm	NA
8	*TaCLPB-5A*	5AL	TraesCS5A02G547300	2925	974	plastid	70
9	*TaCLPB-4B*	4BL	TraesCS4B02G380800	2928	975	plastid	71
10	*TaCLPB-4D*	4DL	TraesCSU02G131300	2917	971	plastid	67
11	*TaCLPB-6A*	6AS	TraesCS6A02G146400	2976	991	mitochondria	85
12	*TaCLPB-6B*	6BS	TraesCS6B02G174500	2976	991	mitochondria	85
13	*TaCLPB-6D*	6DS	TraesCS6D02G135600	2976	991	mitochondria	85

Results from the genome analysis support our hypothesis that at least six family members of CLPB sequences are present in hexaploid wheat, as the diploid and tetraploid progenitors have two and four copies of the gene, respectively^58^. Based on the survey mapping of chromosomal locations, followed by the prediction of subcellular localizations, we found that the three previously reported *TaHSP10I* genes^34,37^ are cytoplasmic CLPBs on the chromosome group1 and 3. We are reporting that homoeologous sequences from the group 1 and 3 are present on A, B, and D chromosomes. Based on our *in silico* mapping, we found that the HSP101 (AF083344.2) that was originally isolated from wheat and functionally characterized by Wells *et al*. (1998) is present on chromosome 1AL. The HSP101 gene previously known as *TaHSP101B* (AF097363.1) is present on chromosome 1DL and *TaHSP101C* (AF174433.1) is present on the chromosome 3DL.

Congruent with the five wheat CLPB copies reported by Muthusamy *et al*. (2016), our analysis showed the presence of eight additional copies clustered into three groups of the cytoplasmic CLPB, one group of the plastid targeted CLPB, and one group of the mitochondria targeted CLPB. Each group consists of three genes that are present on the corresponding three homoeologous chromosomes. Only one of the sequences that was reported (GenBank ID: AK330787) does not match with our sequence annotation. This sequence was previously predicted as a plastid targeted CLPB. This partial sequence is actually located on chromosome 3D, while our predicted sequences of the plastid targeted CLPB are mapped on chromosome 5A, 4B, and 4D.

A *TaCLPB* gene that is present on 4BL does not have any homoeologs on the chromosome 4. Although the wheat genome sequence coverage is good, it is still not clear if the lack of homoeologs for this sequence is real or is simply because of the lack of corresponding sequences in the database. The same results were obtained after we reanalyzed the sequence with the newly released IWGSC RefSeq assembly v 1.0. We did however find a sequence in *Triticum urartu* (EnsemblePlants ID: *TRIUR3_09779*) that appears to be an ortholog of this copy suggesting the presence of the copy A-homoeolog in the progenitor species. Interestingly, the copy in *T. urartu* has a unique insertion in the 5’ end of the mRNA, giving an additional start codon to the sequence. This additional sequence encodes 160 amino acids that contains a transposon domain motif (Supplement 6).

### Structural features of TaCLPBs

**Figure 1:**
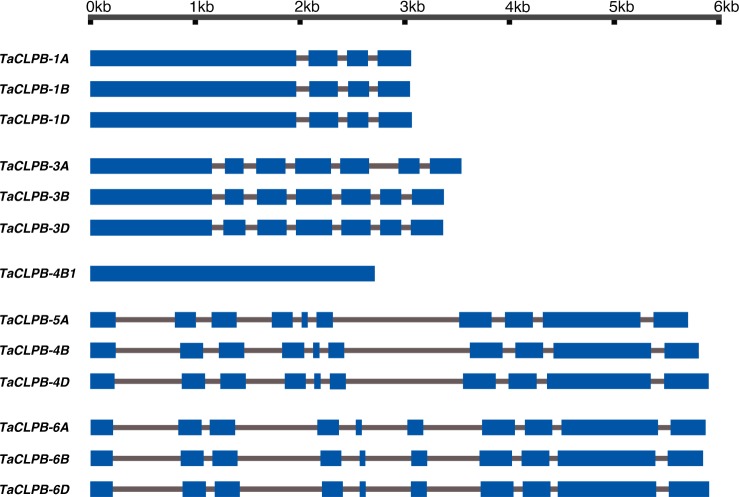
Exon-intron organization of *TaCLPB* genes. The gene structures are drawn from the start codon to the stop codon. Exons and introns are shown in blue and grey, respectively.

**Figure 2:**
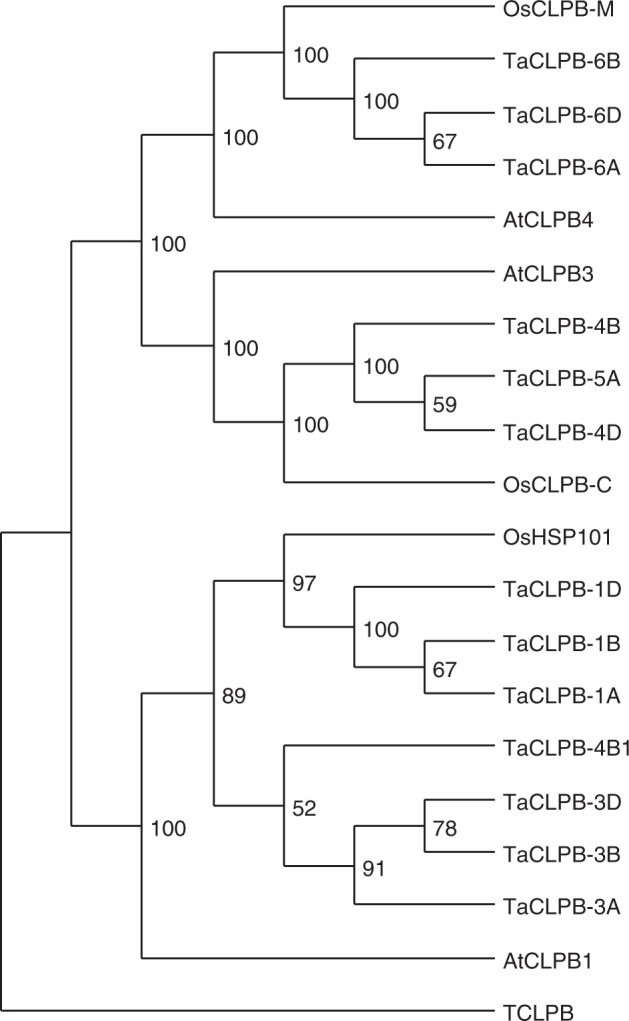
Phylogenetic tree showing relationship among CLPB family in *Arabidopsis*, rice, and wheat. The tree was constructed using a maximum likelihood method in RAxML v.8.2.12 based on the alignment of amino acid sequences.

Structural comparisons of *TaCLPB* genes showed variations in their intron number and size (Fig. 1). While the organellar copies have a higher number of introns than the cytoplasmic ones, no intron is present in *TraesCS4B02G393100* (*TaCLPB-4B1*) sequence. At the protein level, amino acid similarities among TaCLPBs range between 45.4–98.8% (Supplement 7). High similarities were observed among sequences of the same sub-cellular target. There are less sequence similarities between cytoplasmic and organellar CLPBs (46–50%). TraesCS4B02G393100 (TaCLPB-4B1) protein uniquely has the least sequence similarity with the other CLPB members (46–78%). A phylogenetic tree constructed by the maximum likelihood method showed sequence clusters that followed the predicted groups of sub-cellular localizations **(**Fig. 2**)**.

The 3D structures were compared among TaCLPBs and with their orthologs in rice, *Arabidopsis*, and *Thermus thermophilus*. The results showed that structural similarities among CLPB proteins are high (78–93%) regardless of the sequence similarities (Table 2). Interestingly, there are higher structural similarities between TaCLPBs with their corresponding orthologs in *Arabidopsis* although their sequence identities are higher with the orthologs in rice. As an example, TraesCS1A02G340100 (TaCLPB-1A) has 92% identity with the rice ortholog and 84% identity with AtCLPB1, but its 3D structure similarity is 84% with the OsHSP101 and 91% 3D similarity with the AtCLPB1.

**Table 2:** Comparison of TaCLPB protein structures with rice, *Arabidopsis*, and *Thermus thermophilus* CLPB proteins.

Sequence No	Name	OsHSPI01		AtCLPB1		TCLPB	
		*% seq*	*% sse*	*% seq*	*% sse*	*% seq*	*% sse*
1	TaCLPB-1A	92	91	83	91	51	84
2	TaCLPB-1B	93	84	84	91	52	86
3	TaCLPB-1D	92	86	83	93	52	84
4	TaCLPB-3A	92	93	84	91	51	86
5	TaCLPB-3B	91	84	84	93	52	84
6	TaCLPB-3D	91	87	85	87	51	82
7	TaCLPB-4B1	74	85	69	93	49	85
**Sequence** **No**	**Name**	**OsCLPB-C**		**AtCLPB3**		**TCLPB**	
		***% seq***	***% sse***	***% seq***	***% sse***	***% seq***	***% sse***
8	TaCLPB-5A	93	84	82	82	56	80
9	TaCLPB-4B	93	80	84	86	56	78
10	TaCLPB-4D	93	85	83	83	56	85
**Sequence** **No**	**Name**	**OsCLPB-M**		**AtCLPB4**		**TCLPB**	
		***% seq***	***% sse***	***% seq***	***% sse***	***% seq***	***% sse***
11	TaCLPB-6A	91	85	78	89	55	85
12	TaCLPB-6B	92	84	78	80	54	82
13	TaCLPB-6D	90	83	78	87	55	83

% seq = the percentage of sequence identities; % sse = the percentage of protein structure identities.

**Figure 3:**
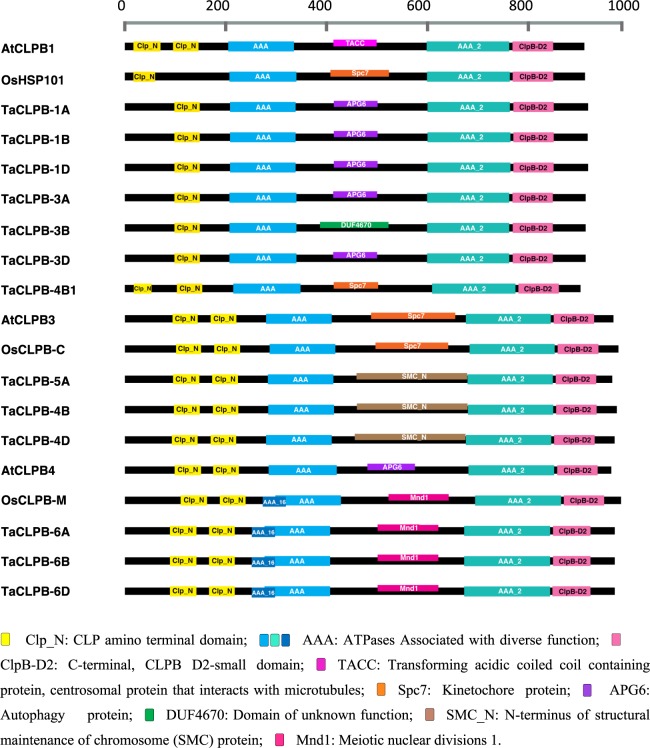
Conserved domain motifs present in CLPB proteins of *Arabidopsis*, rice and wheat.

We referred to *Thermus thermophilus* protein structure^29^ and identified domain conservation across the sequences. Three domain clusters were observed in all the TaCLPB proteins (Fig. 3): (1) Clp_N (Clp amino terminal domain); (2) P-Loop_NTPase (P-Loop containing Nucleoside Triphosphate Hydrolase); and 3) Clp_D2-Small (C-terminal, D2-small domain, of CLPB protein). Two nucleotide binding domains (P-Loop_NTPase) and one C-terminal domain (Clp_D2-Small) were identified in all CLPB proteins. Only one N-domain motif is present in the cytoplasmic chromosome group 1 and group 3 TaCLPB while the other members have two motifs.

In the middle region, which is a coiled coil structure, domain motifs were identified to be varied among the CLPB proteins (Fig. 3). Some motifs are related to the autophagy protein (APG6), and several other motifs are related to the proteins involved in cell divisions (TACC, Spc7, SMC_N, Mnd1). In general, these domain motifs reflect variation that could be present at the functional level, determined by the linker region. The importance of a middle domain for the specificity of CLPB activities has been well studied in yeast and bacterial systems^33^. While other domains were interchangeable in the chimeras of yeast HSP104 and bacterial CLPB, exchanging the middle domain led to a failure in protein function. The middle domain of yeast HSP104 was not able to interact with bacterial DnaK and the middle domain of CLPB could not interact with yeast HSP70. The regions within helix 2 and helix 3 of the middle regions were identified to be responsible for this specificity^33^.

**Figure 4:**
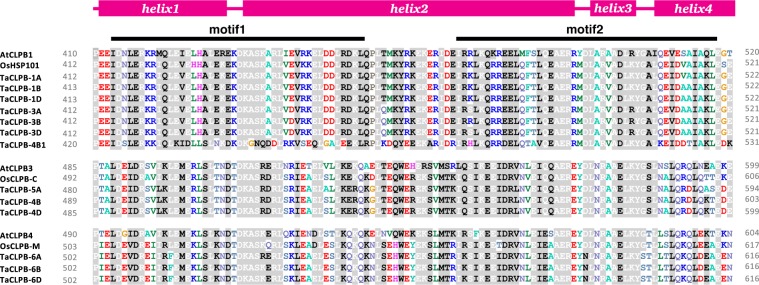
Variation in the residues of middle domain motifs of CLPB proteins in *Arabidopsis*, rice, and wheat. Secondary structures along with motif locations were characterized through protein sequence alignments with the 3D model of *Thermus thermophilus* CLPB using the I-TASSER program. Conserved residues are shown in white color with grey shade, semi-conserved residues are shown in black color with grey shade, non-conserved residues are shown in different colors without shade.

Some studies have also shown the role of middle domains as a molecular toggle that triggers different functions^31,32^. The loop regions, that were marked as motif 1 and motif 2 in the middle domain, were found to be essential for the interaction with trigger factors such as HSP70. We then looked at the area of the CLPB middle domains in *Arabidopsis*, rice, and wheat (Fig. 4). We specifically marked the regions that are aligned with motif 1 and motif 2 of TCLPB from *Thermus thermophillus*. These two motifs were known to be essential for the functionality of CLPB in the bacteria^29^. Higher sequence conservations in the regions were observed among CLPB members of the same subcellular target locations. Only TraesCS4B02G393100 (TaCLPB-4B1) showed less similarity with the other cytoplasmic CLPBs. Some residues were identified to be unique for different plant species with some minor variations being observed among wheat homoeologs. Looking at the data, it is possible that types of molecules or proteins that interact with CLPBs are unique for different subcellular locations, different chromosomal copies, or even different plant species.

**Figure 5:**
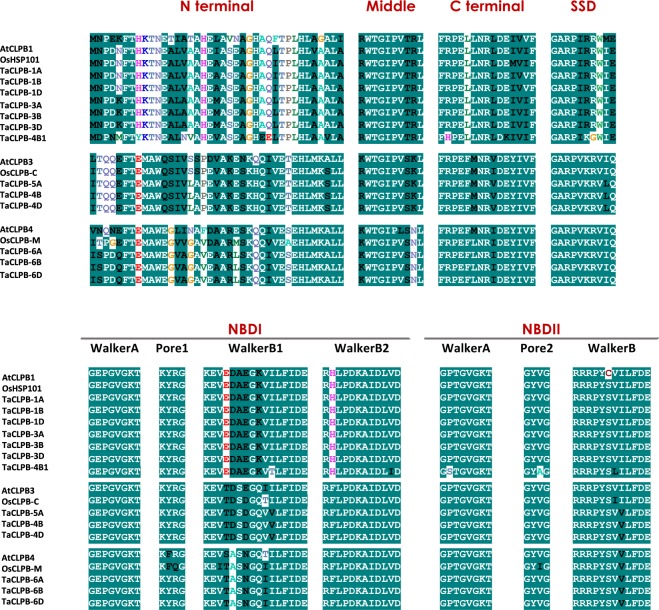
Sequence signatures identified in CLPB sequences of *Arabidopsis*, rice, and wheat. Consensus sequences show signature residues of cytoplasm, plastid and mitochondria targeted CLPBs identified at different conserved regions, including: N-terminal domain, middle domain, C-terminal domain, Sensor and Substrate Discrimination (SSD) motif, Nucleotide binding domain I and II. Consensus residues shown in grey shade share similarity among the three different CLPB proteins. Conserved residues are shown in white color with green shade, semi-conserved residues are shown in black color with green shade, and non-conserved residues are shown in different colors without shade. Positions of residues in the sequences are given in the Supplement 8.

At the sequence level there are signature residues that have been identified in the previous studies and used to differentiate between CLPB with the other ATPase family members^28^. We confirmed the presence of these signatures in all *TaCLPBs* with some minor variations (Fig. 5 and Supplement 8); xKFTxxxxxALAxAxxLAxxxxHxxhxPhHLAxALh at the N-terminus; Gx_4_GKT of Walker A, Kx_6–10_H_4_D of Walker B1, and Rx_6_AIDLHD of Walker B2 at the NBD1; RWTGIPVxKH at the middle domain; GxGKT of Walker A and Rx_6_h_4_D of Walker B at the NBD2; FRPEFLNRLDEIIVFxxL at the C-terminus. We also observed some motifs of KYRG of pore 1, GYVG of pore 2, and GARPHxRxHx of sensor and substrate determination (SSD) that are important for the activity of CLPB^29,74^. Additionally, three unique signatures that are specific for the CLPB proteins were identified at the N-terminus and used to distinguish sequences of different subcellular localizations, they are: MNPxx for cytoplasmic targeted sequences; HTQQE for the plastid targeted sequences; and HSPDx for mitochondria targeted sequences. Since the N-domain is considered to function as a substrate recognition element of the protein^25^, the motifs may indicate variation in the substrates that interact with CLPB members.

**Figure 6:**
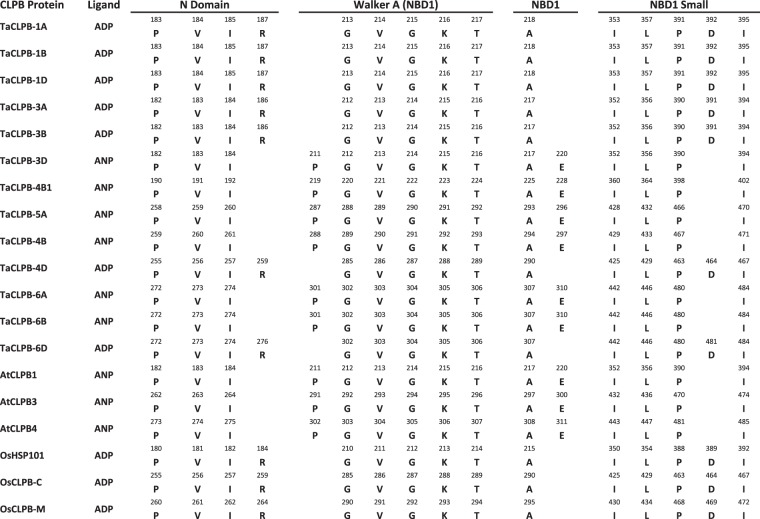
Residues in the predicted ligand binding sites were mapped to the CLPB conserved domains of *Arabidopsis*, rice, and wheat. The single letter amino acids are labelled with their positions in the protein.

To detect interactions of TaCLPB with other molecules, a ligand binding prediction was performed using the COACH program. Several ligand binding sites were identified in the wheat CLPB sequences as shown in the Table 3. Since this prediction relied on the database of conserved ligand binding across species, unique binding sites might not be identified through the analysis. High confidence scores (C-score) were shown by the binding of wheat CLPB proteins to ANP (the analog of ATP) and ADP. We mapped residues of these two binding sites with respect to the position of conserved domains of CLPBs in the Fig. 6. The major binding sites were located at the N-terminal and the first nucleotide-binding domain (NBD1). In yeast, the type of nucleotide ligand was found to regulate the affinity of HSP104 toward polypeptides^75^. It will be interesting to see whether the tendency to bind ADP or ANP ligands control the affinity of plant CLPBs to their substrate polypeptides. Some minor binding sites with a lower C-score were also observed for the CLPB members, including ATP, AF3 (aluminum fluoride), MG (magnesium), and GAI (Guanidine), that have also been reported earlier^76,77^.

**Table 3:** Predicted ligand binding properties of CLPB proteins in wheat, rice, and *Arabidopsis*.

CLPB protein	*Ligand binding prediction in C-score*					
	ADP	ATP	ANP	AF3	MG	GAI
TaCLPB-1A	**0.75** 0.21				0.05	
TaCLPB-1B	**0.77** 0.21			0.03 0.02	0.05	
TaCLPB-1D	**0.77** 0.21	0.01		0.03 0.02	0.05 0.01	
TaCLPB-3A	**0.75** 0.21	0.01			0.05	
TaCLPB-3B	**0.77** 0.21	0.01		0.03	0.05	
TaCLPB-3D	**0.78** 0.20	0.01			0.05	
TaCLPB-4B1	0.22	0.01	**0.77**		0.05 0.01	
TaCLPB-5A	0.19	0.01	**0.75**		0.05	
TaCLPB-4B	0.33	0.01	**0.98**	0.02	0.05	
TaCLPB-4D	**0.74** 0.17			0.03	0.05	
TaCLPB-6A	0.20		**0.73**		0.05	0.02
TaCLPB-6B	0.19		**0.76**	0.03	0.05 0.02	0.02
TaCLPB-6D	**0.76** 0.21	0.01			0.05	0.02
AtCLPB1	**0.93** 0.37				0.05 0.01	
AtCLPB3	**0.93** 0.32	0.01		0.02	0.05	0.02
AtCLPB4	**0.91** 0.33				0.05	
OsHSP101	0.36	0.01	**0.94**	0.02	0.04 0.01	
OsCLPB-C	0.32		**0.92**		0.05 0.01	
OsCLPB-M	0.33	0.01	**0.90**	0.02	0.05	0.02

ADP = Adenosine Diphosphate; ATP = Adenosine Triphosphate; ANP = Phosphoaminephosponic Acid-Adenylate Ester; AF3 = Aluminium Fluoride; MG = Magnesium; GAI = Guanidine.

### Expression analyses of *TaCLPB* genes

Initial expression study of *TaCLPB* genes was performed *in silico* by using publicly available RNA-seq databases. The expression patterns of the family members were studied at different developmental stages, tissues, and abiotic stress (drought and heat) conditions. The *TaCLPB* gene model IDs obtained from Ensemble database were listed in the Table 1.

**Figure 7:**
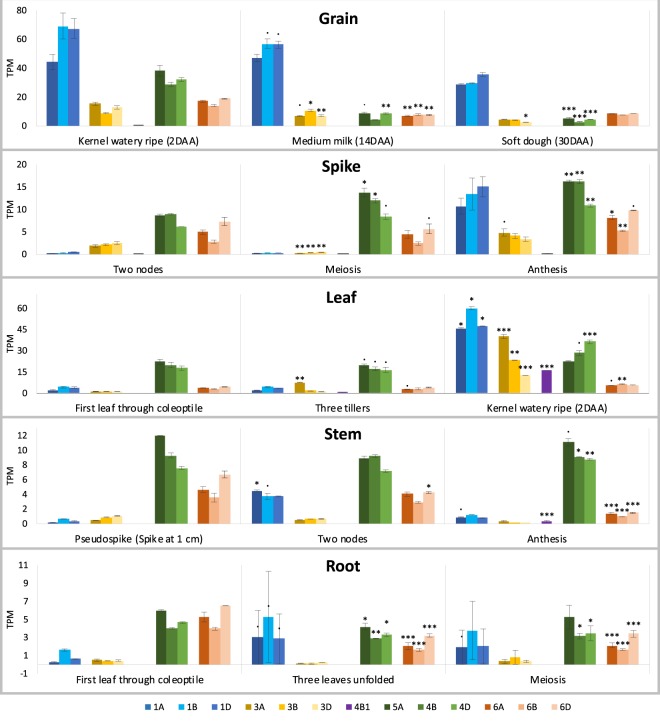
The expression of *TaCLPB* genes in different tissues and life stages. Digital analysis was done using the expVIP wheat expression browser against an RNAseq database of the wheat development time course (*T. aestivum* cv. Chinese Spring; ENA: ERP00471 4). Transcript per million (TPM) is the RNAseq expression unit. Differential expression was analyzed statistically in the EdgeR program. Stars are representing significant difference at one stage in comparison with the earliest stage of each tissue in the analysis (Significance codes: <0.0001 ‘***’; <0.001 ‘**’; <0.01 ‘*’; <0.05 ‘ . ’).

Figure 7 shows *TaCLPB* gene expression in roots, stems, leaves, spikes, and grains at three different life stages following the Zadok’s growth scale. Low expression of *TaCLPBs* were shown in roots and stems. In the leaves, expression increases were observed from the three-tiller stage to 2DAA. Cytoplasmic *TaCLPBs* showed lower expression compared to the organellar members at the vegetative stages but increased significantly after meiosis until the early grain filling stage in leaves and reproductive tissues. *TraesCS4B02G393100* (*TaCLPB-4B1*) expression was observed in mature leaves at the grain filling stage. Meanwhile, organellar targeted genes showed relatively stable expression at all stages, with decreases in reproductive tissues at the later stages. In general, at 30DAA, the expression levels of all *TaCLPBs* were decreased four to eight folds (Supplement 10).

**Figure 8:**
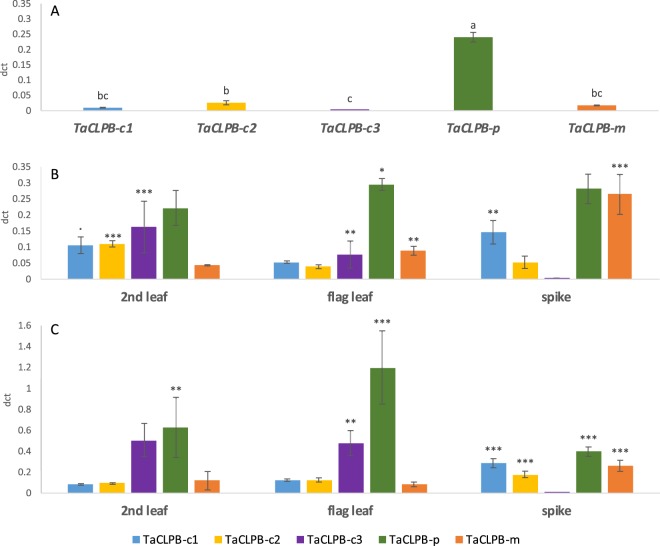
Real time expression of *TaCLPB* genes in different tissues at the seedling (**A**), the anthesis (**B**) and & the 7DAA grain filling (**C**) stages. Five primers were used to amplify transcripts from the cytoplasmic (*TaCLPB-c1, TaCLPB-c2, TaCLPB-c3*), the plastid (*TaCLPB-p*), and the mitochondria (*TaCLPB-m*) groups respectively. Statistics were done using the analysis of variance (anova) for multiway-treatment structure, followed by a post-hoc multiple comparison using Tukey’s test. Letters are indicating statistical variation among genes at the control. Stars are representing significant differences of expression in comparison with the control (Significance codes: <0.0001 ‘***’; <0.001 ‘**’; <0.01 ‘*’; <0.05 ‘ . ’).

Using real-time PCR, we confirmed the expression patterns of *TaCLPB* genes in a group-wise. In total, five primers were designed to amplify *TaCLPBs* of the cytoplasmic (TaCLPB-c) groups from the group 1 chromosomes (TaCLPB-c1), the group 3 chromosomes (TaCLPB-c2), and the group 4 chromosomes (TaCLPB-c3); the plastid targeted group (TaCLPB-p); and the mitochondria targeted group (TaCLPB-m). Variation in expression levels were observed among the *TaCLPB* groups at the seedling, anthesis, and grain filling (7DAA) stages in different tissues including young leaves, mature leaves (second leaves), flag leaves, and spikes (Fig. 8). Similar patterns of group expression were observed between the real-time PCR and the *in silico* analyses. At the seedling stage, except the plastid group, all the *TaCLPB* members showed low expression. Cytoplasmic groups of the chromosome 1 and 3 were more expressed in leaves and reproductive tissues (spike) in the beginning of the reproductive stages (anthesis-7DAA). At these stages, *TraesCS4B02G393100* (*TaCLPB-4B1*) was significantly expressed in the second leaves and flag leaves. Plastid targeted TaCLPB group showed highest expression level in all stages, except in the spike at the 7DAA which tend to be lower. Meanwhile, expression of mitochondrial group was significantly increased in spikes at the anthesis and 7DAA.

Previous studies on the developing plant organs of maize and wheat have revealed HSP101 expression during plant growth and development. Without stress treatments, cytoplasmic HSP101 proteins were abundant in tassels (at the pre-meiosis stage), ears, silks, endosperms, and the embryos of both plants. During kernel imbibition, maize HSP101 decreased and finally disappeared within 3 days^78^. Very little HSP101 protein was present in the leaves and roots under this non-stress condition. However, in maize the level of HSP101 protein and transcript were increased after heat treatments in the vegetative and floral meristematic regions, fully expanded foliar leaves, young ears and roots; but not in anthers at the anthesis, mature pollens, and in the developing endosperm or embryos^79^. We observed similarities between the previous studies with our observation on the cytoplasmic *TaCLPBs* of the chromosomes 1 and 3. It seems the proteins are produced and accumulated during seed formation but not required in the vegetative stage, unless there is a stress.

**Figure 9:**
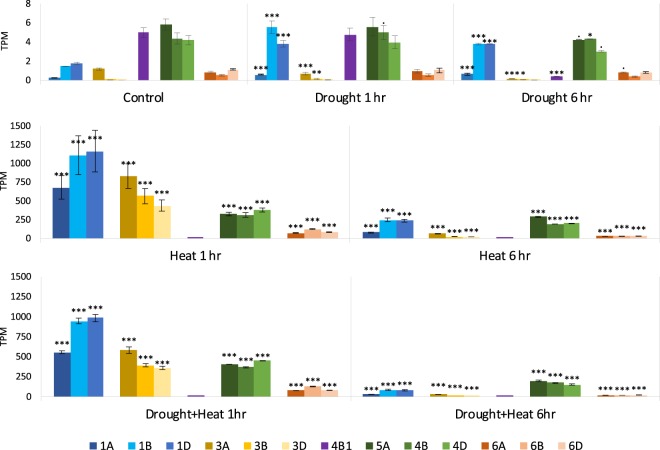
The expression of *TaCLPB* genes under drought and heat treatments. Digital analysis was done using the expVIP wheat expression browser against an RNAseq database of the heat and drought stress (*T, aestivum* cv. TAM107; SRA: SRX25791 5). The RNA libraries were made from leaves of 1-week old seedlings at a control condition (**A**); one-hour and six-hour drought treatments using 20% PEG6000 (**A**); one-hour and six-hour 40 °C heat treatments (**B**); and the combinations of drought and heat treatments (**C**). Transcript per million (TPM) is the RNAseq expression unit. Differential expression was analyzed statistically in the EdgeR program. Stars are representing significant changes by treatments in comparison with the control (Significance codes: <0.0001 ‘***’; <0.001 ‘**’; <0.01 ‘*’; <0.05 ‘ . ’).

*In silico* expression of *TaCLPBs* under drought and heat treatments were shown in the Fig. 9. Under drought treatment. *TaCLPB*s of the chromosome 1 increased expression 2–4 folds without significant differences between 1 h and 6 h treatments (Supplement 11). Expression decreases were observed in *TraesCS3A02G274400* (*TaCLPB-3A*) and *TraesCS4B02G393100* (*TaCLPB-4B1*) by 6 h drought stress. Under heat stress, expression increases were observed in all the *TaCLPB* members, except *TraesCS4B02G393100* (*TaCLPB-4B1*). Five-hour extension of heat stress resulted in lower level of gene expression. Similar patterns were observed under the combination of heat and drought treatments.

The roles of organellar CLPBs in heat tolerance have not been explored in as much detail as the cytoplasmic one. In *Arabidopsis* and rice, potency of the proteins to confer thermotolerance was revealed by the ability of the CLPB genes to complement yeast *hsp104* mutant^14,16,80^. Over expression of cytoplasmic CLPB could improve thermotolerance to 45–50 °C heat stress in rice^81^. In *Arabidopsis*, besides its ability to confer thermotolerance^82^, cytoplasmic CLPB or known as HSP101 was found to have pleiotropic effects which affect plant fitness^83^. Studies on organellar CLPB have been reported in *Arabidopsis* and tomato. Plastid target CLPB was predicted to play a role in chloroplast formation and conferring thermotolerance in this organelle^19^. Silencing of a plastid targeted *CLPB* caused impaired acquisition of thermotolerance in tomato^21^. *In silico*, we observed increases of expression of the organellar CLPBs under heat treatments in leaves at the seeding stage. This increased expression indicates a potential role of organellar CLPBs under the stress, perhaps in collaboration with the cytoplasmic CLPBs.

### Homoeologous specific copies of the Chromosome group 1 *TaCLPBs*

**Figure 10:**
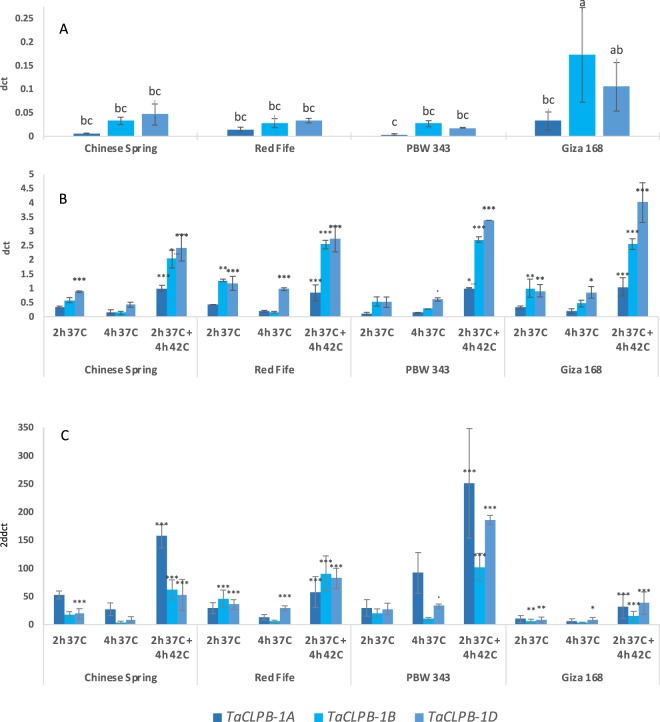
Homoeologous-specific expression of the chromosome I cytoplasmic *TaCLPBs* in four wheat genotypes under different heat treatments. Transcript analysis was done by qPCR using a relative quantification in three biological replicates. 2dct represents a normalized expression ratio (in comparison with actin reference), in the leaves of 12-day old seedlings under 22 °C control (**A**), and heat treatments of 2 h at 37 °C; 4 h at 37 °C; 2 h 37 °C plus 4 h at 42 °C (**B**). 2ddct represents a calibrated expression ratio of transcripts between a treatment and the control. Statistics were done using the analysis of variance (anova) for multiway-treatment structure, followed by a post-hoc multiple comparison using Tukey’s test. Letters are indicating statistical variation among genes at the control. Stars are representing significant changes of expression in comparison with the control (Significance codes: <0.0001 ‘***’; <0.001 ‘**’; <0.01 ‘*’; <0.05 ‘ . ’).

The cytoplasmic members of CLPBs, that are typically known as HSP101, have been well studied in several plant species, including *Arabidopsis*, maize, soybean, and rice^14–16,84^. Most of these studies characterized the gene as a functional ortholog through a yeast *hsp104* complementation, and or by showing its positive effect on thermotolerance. In wheat, a cytoplasmic TaCLPB from the chromosome 1A was known to play a role as an mRNA binding protein that activates protein translations^34^. Though it is still not clear how this role is related to thermotolerance, the ability of the copy to complement yeast *hsp104* indicated its similar function with HSP104. B and D copies, of the chromosome group 1 (*TraesCS1B01G352400* and *TraesCS1D01G342100)* that have not been well characterized, were expected to also share similar functions with yeast HSP104. We confirmed the presence of the A, B, and D homoeologs through sequence cloning and compared their expression under different heat treatments in four wheat genotypes (Fig. 10).

The genomic clones of *TaCLPB*s from the group 1 chromosomes were sequenced and used to confirm the full-length sequences identified through the bioinformatics analysis. To confirm the intron-exon junctions, the three homoeologous copies were aligned with a cDNA sequence of chromosome 1D copy. Minor mistakes observed in the sequences from the database were corrected using these cloned sequences. Homoeologous specific primers were then designed and optimized through a PCR on the Chinese Spring nulli-tetra lines as shown in the Supplement 9. Nulli-tetra lines are the lines that are missing one chromosome pair which is replaced by another homoeologous chromosome pair (nulli 1A means missing a pair of the A homoeologs of the chromosome 1). Using the primers that are designed specifically to amplify the chromosome 1A copy in the PCR, there should be no amplification detected on the nulli 1A (nulli-tetra N1AT1B) template. The same principle is applied for 1B and 1D copy-specific primers, there were no amplification in the PCR on the nulli 1B (nulli-tetra N1BT1D) and nulli 1D (nulli-tetra N1DT1A) templates, respectively.

*In silico* analysis has shown interesting responses of the chromosome 1 *TaCLPBs* under drought and heat treatments. Using real-time PCR, we confirmed this group expression in four wheat genotypes originated from different climate regions (Fig. 10). We found the 1A homoeolog showed significant increase of expression in four genotypes by 2 h 37 °C followed by 4 h 42 °C treatment. Significant increases of 1B homoeolog expression were observed under 2 h 37 °C and the combination of 2 h 37 °C followed by 4 h 42 °C. The 1D homoeolog showed increased expression in all the three treatments in Giza168 and Red Fife. Extended 37 °C heat treatment up to 4 h exposure resulted in a lower expression of *TaCLPB* copies when compared to the shorter 2 h exposure. This is similar with the results from the *in-silico* analysis of the SRA: SRP045409 dataset for the 2 h vs 6 h under 40 °C heat treatments (see Fig. 9), longer exposure to the heat stress decreased *TaCLPB* expression. Decreased gene expression after a long-term heat stress might be related to the optimum level of the CLPB proteins that created a negative feedback loop to the transcript expression.

The variation in expression among homoeologous copies indicates necessity to identify all the homoeologs before studying the gene expression in polyploids. A bias in the expression level could be easily introduced to the analysis by using the primers that are not specific to only one copy, or otherwise common to represent all the homoeologs. This aspect is critical especially if one need to compare the gene expression among genotypes.

In conclusions, complexity of the wheat genome creates special challenges to study gene function and its potential use in breeding programs. In this study, a systematic approach was taken to understand the role of *HSP101*/*CLPB* genes in heat tolerance through a genome-wide bioinformatics analysis, followed by real-time expression studies. Thirteen copies of *CLPB* genes were identified and characterized for their structural variations and differential expression patterns. The results suggest possible different functions of TaCLPBs with respect to their chromosomal and subcellular localizations. The expression analysis of *TaCLPBs* of the group 1 chromosomes revealed variation among homoeologous copies with respect to different temperature treatments. In this experiment, the variation of *TaCLPB* expression among four genotypes would not be enough to show the link between *TaCLPB* with thermotolerance in varieties. Nevertheless, it provides basic information for further studies to reveal the potentials of *TaCLPB* for variety improvement, including development of gene-based markers.

## Supplementary information

Supplementary materials.
