# Supplementary material for: Genome-wide analysis of the HSP101/CLPB gene family for heat tolerance in hexaploid wheat
Source: Sci Rep. 2020 Mar 3;10:3948. doi: 10.1038/s41598-020-60673-4 (PMC7054433; doi:10.1038/s41598-020-60673-4)
Supplement: Supplementary file 1 — Supplementary materials. [file 41598_2020_60673_MOESM1_ESM.pdf]

Genome-wide analysis of the *HSP101/CLPB* gene family for heat tolerance in hexaploid wheat

Authors:

Eva Erdayani[1,2], Ragupathi Nagarajan[1], Nathan P. Grant[1], and Kulvinder S. Gill[1*]

1. Department of Crop and Soil Sciences, Washington State University, Pullman, WA. USA.
2. Research Center for Biotechnology, Indonesian Institute of Sciences, Cibinong, Jawa Barat, Indonesia.

***Corresponding Author:**

Kulvinder S. Gill,

Dept. of Crop and Soil Science, Washington State University. 100 Dairy Road, Johnson Hall 277. Pullman, WA-99164, USA.

Email: ksgill@wsu.edu. Phone: +1509-335-4666. Fax: 509-335-8674

Supplement 1 List of primers

No	Target	Forward primer (5'-3')	Reverse primer (5'-3')	Amplicon size (bp)
**1.**	*TaCLPB-1A* (full-length)	GGGGACAAGTTTGTACAAAAAAGCAGGCTCCAACCCAAAGTCTCTGCCC	GGGGACCACTTTGTACAAGAAAGCTGGGTCAAGCTTCACTTCAGCTTCAGAG	3149
**2.**	*TaCLPB-1B* (full-length)	GGGGACAAGTTTGTACAAAAAAGCAGGCTGTCTGTCTGATCCATTGTAGGTT	GGGGACCACTTTGTACAAGAAAGCTGGGTCAAGCTTCACTTCAGCTTCAGAG	3130
**3.**	*TaCLPB-1D* (full-length)	GGGGACAAGTTTGTACAAAAAAGCAGGCTGTCAGTTCGATCCATTGTAGAGC	GGGGACCACTTTGTACAAGAAAGCTGGGTCAAGCTTCACTTCAGCTTCAGAG	3137
**4.**	*TaCLPB-1A* (partial)	AACAAGGACGAGCTGGCCTA	CAACCATCTTCACTCACAGAGCAC	305
**5.**	*TaCLPB-1B* (partial)	AACAAGGACGAGCTGGCCTA	ACACGCGTCACAGAGCCT	280
**6.**	*TaCLPB-1D* (partial)	AACAAGGACGAGCTGGCCTA	CGCGTCACAGAACAACACG	283
**7.**	*TaCLPB-c1*	GGTGGTGACGCAGCTGT	CCGTTGGGCACCTGGAT	174
**8.**	*TaCLPB-c2*	CACGGAGGGCTGGTGAAC	CATCTACTCTTCCTCCATGTCGTC	156
**9.**	*TaCLPB-c3*	ATGGAGCAAGCTCACGGTAA	CAGGGCGTCGTCAGTGAT	201
**10.**	*TaCLPB-p*	GCGGGTGCTTCAGCAGTA	CTGTCGGCAGGAACTTCTCAT	203
**11.**	*TaCLPB-m*	GTCAAGAGGGTGATCCAGCAG	TGGCAACCAACTCTGTGCTT	184

Primer pair No. 1-3 were used in cloning, No. 4-11 were used in qRT-PCR. The sequences of *attb* sites are marked by the underlines

Supplement 2 Alignment of the cytoplasmic wheat CLPBs (TaCLPB) with rice cytoplasmic HSP101 (OsHSP101). Percent identities between the sequences with the reference are shown at the right side of each sequence name. Color codes indicate conservation of amino acid properties.

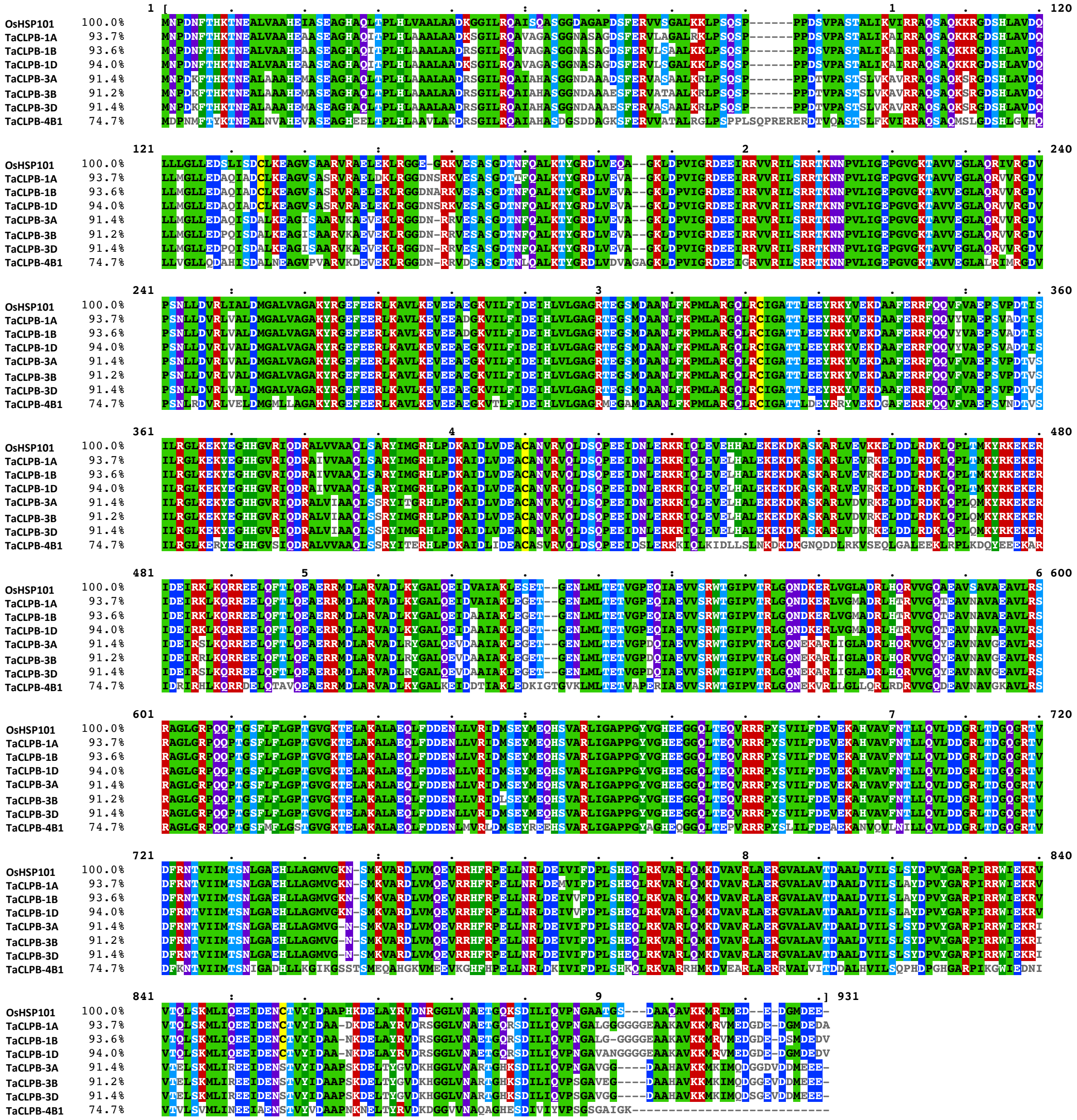

Supplement 3 Alignment of the plastid targeted wheat CLPBs (TaCLPB) with rice CLASS I CLP ATPASE B-C (OsCLPB-C). Percent identities between the sequences with the reference are shown at the right side of each sequence name. Color codes indicate conservation of the amino acid properties.

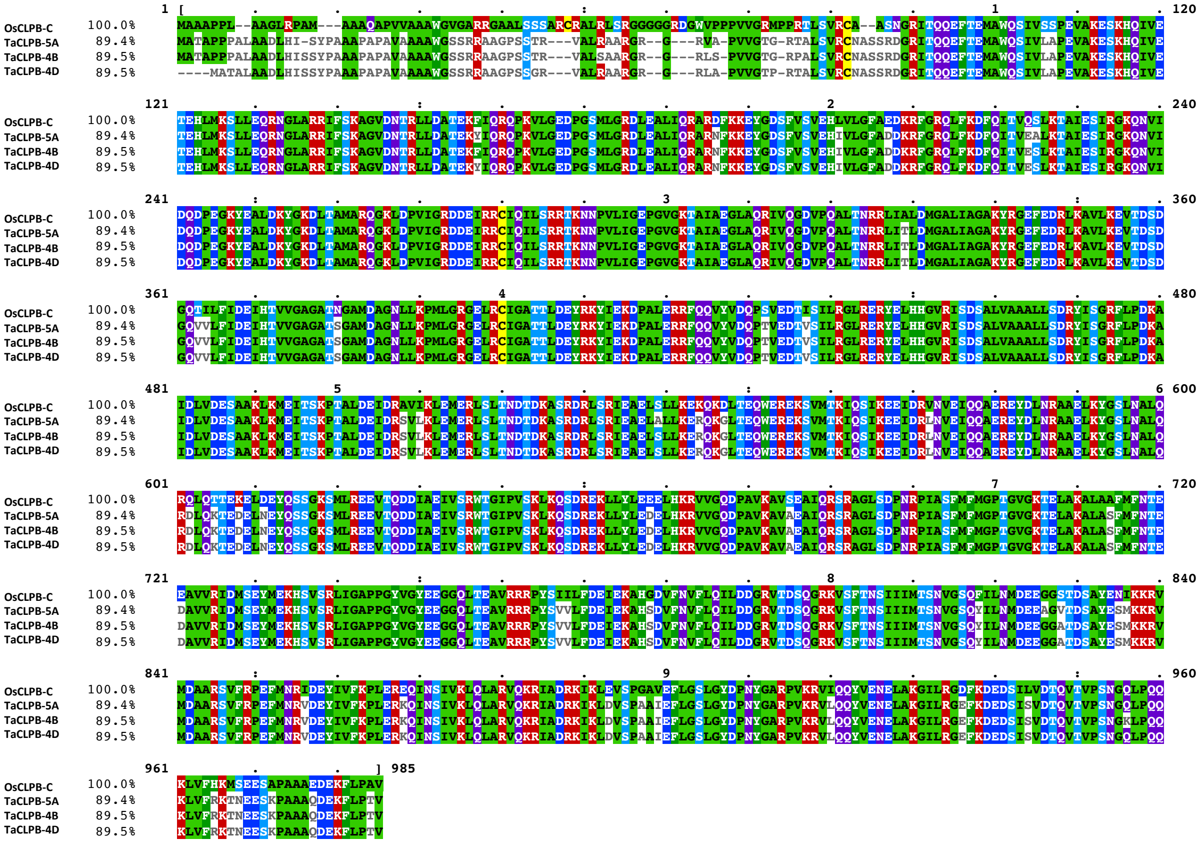

Supplement 4 Alignment of the mitochondria targeted wheat CLPBs (TaCLPB) with the rice CLASS I CLP ATPASE B-M (OsCLPB-M). Percent identities between the sequences with the reference are shown at the right side of each sequence name. Color codes indicate conservation of the amino acid properties.

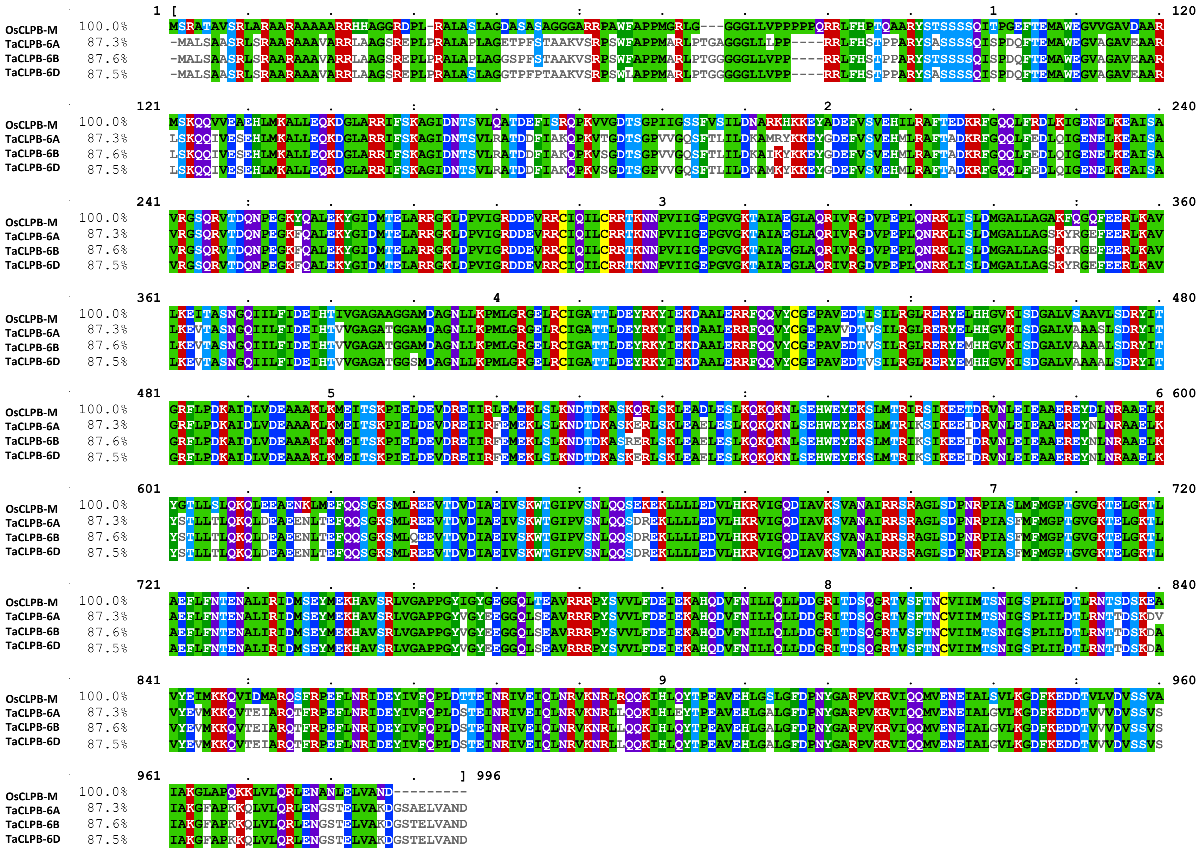

Supplement 5 Synteny of *TaCLPB* genes with the orthologs from other species

Wheat CLPB gene	Species	Type	Orthologue	dN/dS	Target %id	Query %id	GOC Score	High Confidence
TraesCS1A01G340100	Brachypodium distachyon	1-to-1	BRADI_2g19540v3 (2:17,196,536-17,200,270:1)s	n/a	96.71%	96.08%	25	Yes
	Hordeum vulgare	1-to-1	HORVU1Hr1G076190 (chr1H:513,272,813-513,276,751:-1)s	0.01242	98.47%	98.26%	50	Yes
	Oryza sativa Indica	1-to-1	BGIOSGA017714 (5:27,252,159-27,255,267:-1)s	0.0265	94.19%	93.68%	n/a	Yes
	Oryza sativa Japonica	1-to-1	HEAT SHOCK PROTEIN 101 (Os05g0519700) (5:25,803,609-25,807,160:-1)s	0.02652	94.30%	93.68%	n/a	Yes
	Setaria italica	1-to-1	SETIT_021144mg (III:13,015,769-13,019,449:1)s	0.01945	93.76%	93.25%	n/a	Yes
	Sorghum bicolor	1-to-1	SORBI_3009G201500 (9:55,096,931-55,101,127:-1)s	0.01979	93.65%	93.14%	n/a	Yes
	Triticum urartu	1-to-1	TRIUR3_12605 (scaffold1211:8,812-12,599:1)s	0.17004	96.44%	47.28%	0	Yes
	Zea mays	1-to-1	heat-shock protein 101 (Zm00001d038806) (6:164,808,309-164,812,048:1)s	0.01793	93.09%	92.48%	n/a	Yes
TraesCS1B01G352400	Brachypodium distachyon	1-to-1	BRADI_2g19540v3 (2:17,196,536-17,200,270:1)s	0.01303	97.26%	96.73%	25	Yes
	Hordeum vulgare	1-to-1	HORVU1Hr1G076190 (chr1H:513,272,813-513,276,751:-1)s	0.00814	98.91%	98.80%	50	Yes
	Oryza sativa Indica	1-to-1	BGIOSGA017714 (5:27,252,159-27,255,267:-1)s	0.02714	94.19%	93.78%	n/a	Yes
	Oryza sativa Japonica	1-to-1	HEAT SHOCK PROTEIN 101 (Os05g0519700) (5:25,803,609-25,807,160:-1)s	0.02716	94.30%	93.78%	n/a	Yes
	Setaria italica	1-to-1	SETIT_021144mg (III:13,015,769-13,019,449:1)s	0.02002	94.09%	93.67%	n/a	Yes
	Sorghum bicolor	1-to-1	SORBI_3009G201500 (9:55,096,931-55,101,127:-1)s	0.02079	93.98%	93.57%	n/a	Yes
	Triticum urartu	1-to-1	TRIUR3_12605 (scaffold1211:8,812-12,599:1)s	0.0567	96.00%	47.11%	25	Yes
	Zea mays	1-to-1	heat-shock protein 101 (Zm00001d038806) (6:164,808,309-164,812,048:1)s	0.01816	93.31%	92.80%	n/a	Yes
TraesCS1D01G342100	Brachypodium distachyon	1-to-1	BRADI_2g19540v3 (2:17,196,536-17,200,270:1)s	0.01253	97.81%	97.17%	25	Yes
	Hordeum vulgare	1-to-1	HORVU1Hr1G076190 (chr1H:513,272,813-513,276,751:-1)s	0.00594	99.24%	99.02%	50	Yes
	Oryza sativa Indica	1-to-1	BGIOSGA017714 (5:27,252,159-27,255,267:-1)s	0.02617	94.63%	94.12%	n/a	Yes
	Oryza sativa Japonica	1-to-1	HEAT SHOCK PROTEIN 101 (Os05g0519700) (5:25,803,609-25,807,160:-1)s	0.02615	94.74%	94.12%	n/a	Yes
	Setaria italica	1-to-1	SETIT_021144mg (III:13,015,769-13,019,449:1)s	0.01953	94.41%	93.90%	n/a	Yes
	Sorghum bicolor	1-to-1	SORBI_3009G201500 (9:55,096,931-55,101,127:-1)s	0.01947	94.30%	93.79%	n/a	Yes
	Triticum urartu	1-to-1	TRIUR3_12605 (scaffold1211:8,812-12,599:1)s	0.04477	96.22%	47.17%	25	Yes
	Zea mays	1-to-1	heat-shock protein 101 (Zm00001d038806) (6:164,808,309-164,812,048:1)s	0.01816	93.64%	93.03%	n/a	Yes
TraesCS3A01G274400	Brachypodium distachyon	1-to-1	BRADI_2g49660v3 (2:49,478,053-49,482,033:1)s	0.03039	95.18%	95.18%	50	Yes
	Hordeum vulgare	1-to-1	HORVU3Hr1G071120 (chr3H:538,150,623-538,154,544:-1)s	0.03614	97.48%	97.59%	25	Yes
	Setaria italica	1-to-1	SETIT_000232mg (V:36,657,855-36,661,530:1)s	0.04399	92.12%	92.22%	n/a	Yes
	Sorghum bicolor	1-to-1	SORBI_3003G293500 (3:62,597,578-62,601,312:1)s	0.04604	90.38%	90.58%	n/a	Yes
	Triticum urartu	1-to-1	TRIUR3_29568 (scaffold41385:22,928-26,130:1)s	0.96774	99.40%	72.51%	0	Yes
TraesCS3B01G308100	Brachypodium distachyon	1-to-many	BRADI_2g49660v3 (2:49,478,053-49,482,033:1)s	0.0356	95.18%	95.18%	50	Yes
	Hordeum vulgare	1-to-1	HORVU3Hr1G071120 (chr3H:538,150,623-538,154,544:-1)s	0.04021	97.37%	97.48%	25	Yes
	Setaria italica	1-to-many	SETIT_000232mg (V:36,657,855-36,661,530:1)s	0.04594	92.12%	92.22%	n/a	Yes
	Sorghum bicolor	1-to-many	SORBI_3003G293500 (3:62,597,578-62,601,312:1)s	0.05098	90.16%	90.36%	n/a	Yes
	Triticum urartu	1-to-1	TRIUR3_29568 (scaffold41385:22,928-26,130:1)s	0.04197	98.35%	71.74%	0	Yes
TraesCS3D01G273600	Brachypodium distachyon	1-to-1	BRADI_2g49660v3 (2:49,478,053-49,482,033:1)s	0.03411	95.29%	95.29%	50	Yes
	Hordeum vulgare	1-to-1	HORVU3Hr1G071120 (chr3H:538,150,623-538,154,544:-1)s	0.03324	97.70%	97.81%	25	Yes
	Setaria italica	1-to-1	SETIT_000232mg (V:36,657,855-36,661,530:1)s	0.04868	92.23%	92.33%	n/a	Yes
	Sorghum bicolor	1-to-1	SORBI_3003G293500 (3:62,597,578-62,601,312:1)s	0.05397	90.27%	90.47%	n/a	Yes
	Triticum urartu	1-to-1	TRIUR3_29568 (scaffold41385:22,928-26,130:1)s	0.02806	98.65%	71.96%	0	Yes
TraesCS4B01G393100	Brachypodium distachyon	1-to-many	BRADI_2g49660v3 (2:49,478,053-49,482,033:1)s	0.25262	76.01%	76.85%	0	Yes
	Setaria italica	1-to-many	SETIT_000232mg (V:36,657,855-36,661,530:1)s	0.16042	74.51%	75.42%	n/a	Yes
	Sorghum bicolor	1-to-many	SORBI_3003G293500 (3:62,597,578-62,601,312:1)s	0.1621	73.11%	74.09%	n/a	Yes
	Triticum urartu	1-to-1	TRIUR3_0977 9 (scaffold42502:77,026-81,719:1)s	0.11864	82.60%	93.58%	25	Yes
TraesCS5A01G547300	Brachypodium distachyon	1-to-1	BRADI_1g16190v3 (1:13,104,612-13,112,560:-1)s	0.08882	94.23%	93.84%	0	Yes
	Hordeum vulgare	1-to-1	HORVU4Hr1G090440 (chr4H:645,151,567-645,157,915:1)s	0.08148	89.89%	97.64%	75	Yes
	Oryza sativa Indica	1-to-1	BGIOSGA012917 (3:19,973,224-19,979,567:1)s	0.07785	91.39%	90.45%	n/a	Yes
	Oryza sativa Japonica	1-to-1	CLASS I CLP ATPASE B-C (Os03g0426900) (3:17,832,202-17,839,082:1)s	0.08044	90.80%	91.17%	n/a	Yes
	Setaria italica	1-to-1	SETIT_034086mg (IX:42,170,576-42,176,727:-1)s	0.07778	90.17%	90.45%	n/a	Yes
	Sorghum bicolor	1-to-1	SORBI_3001G333500 (1:62,224,868-62,233,524:-1)s	0.07137	89.52%	90.35%	n/a	Yes
	Triticum urartu	1-to-1	TRIUR3_26934 (scaffold108158:11,107-16,002:1)s	0.08108	99.89%	90.97%	0	Yes
	Zea mays	1-to-many	Zm00001d029557 (1:76,065,155-76,078,721:1)s	0.06873	90.08%	90.45%	n/a	Yes
	Zea mays	1-to-many	Zm00001d047302 (9:126,065,827-126,072,415:1)s	0.06831	90.25%	90.25%	n/a	Yes
TraesCS4B01G380800	Brachypodium distachyon	1-to-1	BRADI_1g16190v3 (1:13,104,612-13,112,560:-1)s	0.08391	94.23%	93.74%	0	Yes
	Hordeum vulgare	1-to-1	HORVU4Hr1G090440 (chr4H:645,151,567-645,157,915:1)s	0.08233	89.89%	97.54%	25	Yes
	Oryza sativa Indica	1-to-1	BGIOSGA012917 (3:19,973,224-19,979,567:1)s	0.07211	91.60%	90.56%	n/a	Yes
	Oryza sativa Japonica	1-to-1	CLASS I CLP ATPASE B-C (Os03g0426900) (3:17,832,202-17,839,082:1)s	0.07486	91.00%	91.28%	n/a	Yes
	Setaria italica	1-to-1	SETIT_034086mg (IX:42,170,576-42,176,727:-1)s	0.07275	90.38%	90.56%	n/a	Yes
	Sorghum bicolor	1-to-1	SORBI_3001G333500 (1:62,224,868-62,233,524:-1)s	0.06726	89.42%	90.15%	n/a	Yes
	Triticum urartu	1-to-1	TRIUR3_26934 (scaffold108158:11,107-16,002:1)s	0.03107	99.21%	90.26%	0	Yes
	Zea mays	1-to-many	Zm00001d029557 (1:76,065,155-76,078,721:1)s	0.0634	89.98%	90.26%	n/a	Yes
	Zea mays	1-to-many	Zm00001d047302 (9:126,065,827-126,072,415:1)s	0.06441	90.14%	90.05%	n/a	Yes
TraesCSU01G131300	Brachypodium distachyon	1-to-1	BRADI_1g16190v3 (1:13,104,612-13,112,560:-1)s	0.0816	94.64%	94.54%	0	Yes
	Hordeum vulgare	1-to-1	HORVU4Hr1G090440 (chr4H:645,151,567-645,157,915:1)s	0.07624	90.08%	98.15%	50	Yes
	Oryza sativa Indica	1-to-1	BGIOSGA012917 (3:19,973,224-19,979,567:1)s	0.07644	91.81%	91.14%	n/a	Yes
	Oryza sativa Japonica	1-to-1	CLASS I CLP ATPASE B-C (Os03g0426900) (3:17,832,202-17,839,082:1)s	0.07891	91.00%	91.66%	n/a	Yes
	Setaria italica	1-to-1	SETIT_034086mg (IX:42,170,576-42,176,727:-1)s	0.07724	90.28%	90.83%	n/a	Yes
	Sorghum bicolor	1-to-1	SORBI_3001G333500 (1:62,224,868-62,233,524:-1)s	0.0714	89.52%	90.63%	n/a	Yes
	Triticum urartu	1-to-1	TRIUR3_26934 (scaffold108158:11,107-16,002:1)s	0.02473	99.44%	90.83%	0	Yes
	Zea mays	1-to-many	Zm00001d029557 (1:76,065,155-76,078,721:1)s	0.06598	90.08%	90.73%	n/a	Yes
	Zea mays	1-to-many	Zm00001d047302 (9:126,065,827-126,072,415:1)s	0.06534	90.25%	90.53%	n/a	Yes
TraesCS6A01G146400	Brachypodium distachyon	1-to-1	BRADI_3g06107v3 (3:4,408,352-4,414,831:-1)s	0.10532	92.91%	92.53%	25	Yes
	Hordeum vulgare	1-to-1	HORVU6Hr1G028690 (chr6H:114,383,996-114,391,210:-1)s	0.12072	97.26%	96.57%	0	Yes
	Oryza sativa Indica	1-to-1	BGIOSGA007016 (2:5,308,253-5,315,863:-1)s	0.09803	71.53%	87.99%	n/a	Yes
	Oryza sativa Japonica	1-to-1	CLASS I CLP ATPASE B-M (Os02g0181900) (2:4,567,630-4,573,049:-1)s	0.09621	88.81%	88.09%	n/a	Yes
	Setaria italica	1-to-1	SETIT_016214mg (I:5,798,068-5,804,551:1)s	0.10964	88.88%	88.70%	n/a	Yes
	Sorghum bicolor	1-to-1	SORBI_3004G066500 (4:5,408,847-5,416,471:-1)s	0.09917	88.48%	88.40%	n/a	Yes
	Triticum urartu	1-to-1	TRIUR3_24739 (scaffold8792:23,055-28,816:-1)s	0.32075	98.31%	93.84%	25	Yes
	Zea mays	1-to-1	Zm00001d015520 (5:95,614,609-95,620,842:-1)s	0.09812	88.46%	88.19%	n/a	Yes
TraesCS6B01G174500	Brachypodium distachyon	1-to-1	BRADI_3g06107v3 (3:4,408,352-4,414,831:-1)s	0.10489	92.81%	92.43%	25	Yes
	Hordeum vulgare	1-to-1	HORVU6Hr1G028690 (chr6H:114,383,996-114,391,210:-1)s	0.12312	97.05%	96.37%	0	Yes
	Oryza sativa Indica	1-to-1	BGIOSGA007016 (2:5,308,253-5,315,863:-1)s	0.09828	71.21%	87.59%	n/a	Yes
	Oryza sativa Japonica	1-to-1	CLASS I CLP ATPASE B-M (Os02g0181900) (2:4,567,630-4,573,049:-1)s	0.09649	88.40%	87.69%	n/a	Yes
	Setaria italica	1-to-1	SETIT_016214mg (I:5,798,068-5,804,551:1)s	0.10664	88.57%	88.40%	n/a	Yes
	Sorghum bicolor	1-to-1	SORBI_3004G066500 (4:5,408,847-5,416,471:-1)s	0.0965	88.18%	88.09%	n/a	Yes
	Triticum urartu	1-to-1	TRIUR3_24739 (scaffold8792:23,055-28,816:-1)s	0.1831	97.25%	92.84%	25	Yes
	Zea mays	1-to-1	Zm00001d015520 (5:95,614,609-95,620,842:-1)s	0.09484	88.16%	87.89%	n/a	Yes
TraesCS6D01G135600	Brachypodium distachyon	1-to-1	BRADI_3g06107v3 (3:4,408,352-4,414,831:-1)s	0.10367	92.91%	92.53%	25	Yes
	Hordeum vulgare	1-to-1	HORVU6Hr1G028690 (chr6H:114,383,996-114,391,210:-1)s	0.121	97.36%	96.67%	0	Yes
	Oryza sativa Indica	1-to-1	BGIOSGA007016 (2:5,308,253-5,315,863:-1)s	0.09798	71.53%	87.99%	n/a	Yes
	Oryza sativa Japonica	1-to-1	CLASS I CLP ATPASE B-M (Os02g0181900) (2:4,567,630-4,573,049:-1)s	0.09598	88.81%	88.09%	n/a	Yes
	Setaria italica	1-to-1	SETIT_016214mg (I:5,798,068-5,804,551:1)s	0.10784	88.78%	88.60%	n/a	Yes
	Sorghum bicolor	1-to-1	SORBI_3004G066500 (4:5,408,847-5,416,471:-1)s	0.09647	88.59%	88.50%	n/a	Yes
	Triticum urartu	1-to-1	TRIUR3_24739 (scaffold8792:23,055-28,816:-1)s	0.09767	98.20%	93.74%	25	Yes
	Zea mays	1-to-1	Zm00001d015520 (5:95,614,609-95,620,842:-1)s	0.09762	88.36%	88.09%	n/a	Yes

Supplement 6 The protein structure of *TraesCS4B02G393100* (A) homolog in *T. Urartu, TRIUR3_09779* (B).

Clp_N = CLP amino terminal domain; AAA = ATPases Associated with diverse function;

ClpB-D2 = C-terminal, CLPB D2-small domain; Spc7 = kinetochore protein;

DDE = DDE superfamily endonuclease; Atg14 = Autophagy related subunit 14

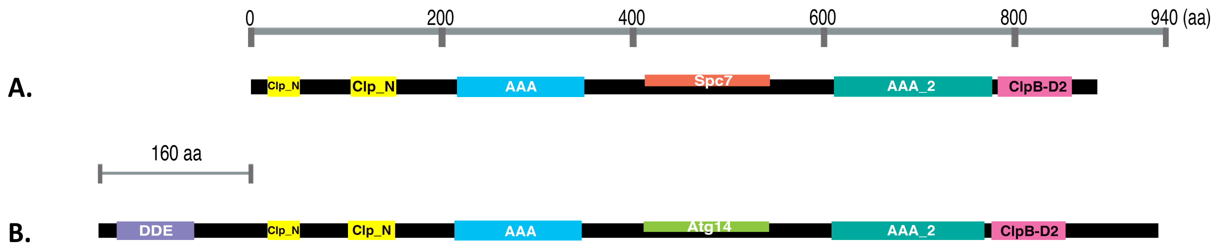

Supplement 7 Identities (%) among TaCLPBs at the protein level

**TaCLPB Protein**	**No.**		**1**	**2**	**3**	**4**	**5**	**6**	**7**	**8**	**9**	**10**	**11**	**12**	**13**
		**Map**	**1AL**	**1BL**	**1DL**	**3AL**	**3BL**	**3DL**	**4BL**	**5AL**	**4BL**	**4DL**	**6AS**	**6BS**	**6DS**
TaCLPB-1A	**1**	**1AL**	100	98	98	94	94	94	77	55	56	56	46	48	47
TaCLPB-1B	**2**	**1BL**		100	98	94	94	95	74	56	57	57	46	49	47
TaCLPB-1D	**3**	**1DL**			100	93	93	94	77	46	48	47	46	48	47
TaCLPB-3A	**4**	**3AL**				100	99	99	78	56	57	57	47	49	47
TaCLPB-3B	**5**	**3BL**					100	99	77	56	57	57	46	48	47
TaCLPB-3D	**6**	**3DL**						100	79	56	57	57	47	49	47
TaCLPB-4B1	**7**	**4BL**							100	53	54	54	44	46	45
TaCLPB-5A	**8**	**5AL**								100	97	97	60	62	61
TaCLPB-4B	**9**	**4BL**									100	98	60	62	61
TaCLPB-4D	**10**	**4DL**										100	60	62	61
TaCLPB-6A	**11**	**6AS**											100	90	90
TaCLPB-6B	**12**	**6BS**												100	94
TaCLPB-6D	**13**	**6DS**													100

Supplement 8 Positions of signature sequences at different conserved regions in CLPB proteins of *Arabidopsis*, rice, and wheat

CLPB protein	N	Middle	C	SSD	NBD1				NBD2		
					WalkerA	Pore1	WalkerB1	WalkerB2	WalkerA	Pore2	WalkerB
AtCLPB1	1-39	543-552	751-765	815-824	207-214	251-254	265-280	385-396	606-613	653-656	668-679
OsHSP101	1-39	544-553	752-766	797-825	209-216	253-256	267-282	387-398	607-614	654-657	669-680
TaCLPB-1A	1-39	555-554	753-767	798-826	210-217	254-257	268-283	388-399	608-615	655-658	670-681
TaCLPB-1B	1-39	555-554	753-767	798-826	210-217	254-257	268-283	388-399	608-615	655-658	670-681
TaCLPB-1D	1-39	555-554	753-767	798-826	210-217	254-257	268-283	388-399	608-615	655-658	670-681
TaCLPB-3A	1-39	544-553	751-765	815-824	209-216	253-256	267-282	387-398	607-614	654-657	669-680
TaCLPB-3B	1-39	544-553	751-765	815-824	209-216	253-256	267-282	387-398	607-614	654-657	669-680
TaCLPB-3D	1-39	544-553	751-765	815-824	209-216	253-256	267-282	387-398	607-614	654-657	669-680
TaCLPB-4B1	1-39	554-563	763-777	827-836	217-224	261-264	275-290	395-406	617-624	664-667	679-690
AtCLPB3	76-115	622-631	833-847	897-906	282-289	326-329	340-355	460-471	685-692	732-735	747-758
OsCLPB-C	83-122	629-638	841-855	905-914	289-296	333-336	347-362	467-478	692-699	739-742	754-765
TaCLPB-5A	79-118	625-634	837-851	901-910	285-292	329-332	343-358	463-474	688-695	735-738	750-761
TaCLPB-4B	80-119	626-635	838-852	902-911	286-293	330-333	344-359	464-475	689-696	736-739	751-762
TaCLPB-4D	76-115	622-631	834-848	898-907	282-289	326-329	340-355	460-471	685-692	727-730	747-758
AtCLPB4	82-121	627-636	840-854	904-913	287-294	331-334	345-360	465-476	690-897	737-740	752-763
OsCLPB-M	94-133	640-649	853-867	917-926	300-307	344-347	358-373	478-489	703-710	750-753	765-776
TaCLPB-6A	93-132	639-648	852-866	916-925	299-306	343-346	357-372	477-488	702-709	749-752	764-775
TaCLPB-6B	93-132	639-648	852-866	916-925	299-306	343-346	357-372	477-488	702-709	749-752	764-775
TaCLPB-6D	93-132	639-648	852-866	916-925	299-306	343-346	357-372	477-488	702-709	749-752	764-775

Supplement 9 Fragment amplification by using homoeologous specific primers of the chromosome 1 *TaCLPB*. M=100 bp ladder; N1AT1B, N1B1D, N1D1A = PCR product using the Chinese Spring wheat Nulli-Tetra lines that are lacking chromosome 1A, 1B, and 1D respectively; (-) = negative control; (+) = positive control. Copy A/B/D primers = PCR product by 1AL/1BL/1DL copy-specific primer (No. 4,5,6 in the **Supplement 1**). Top and bottom lanes are for the same types of samples from two different sets of PCR reaction. Samples were run in 1.5% agarose gel.

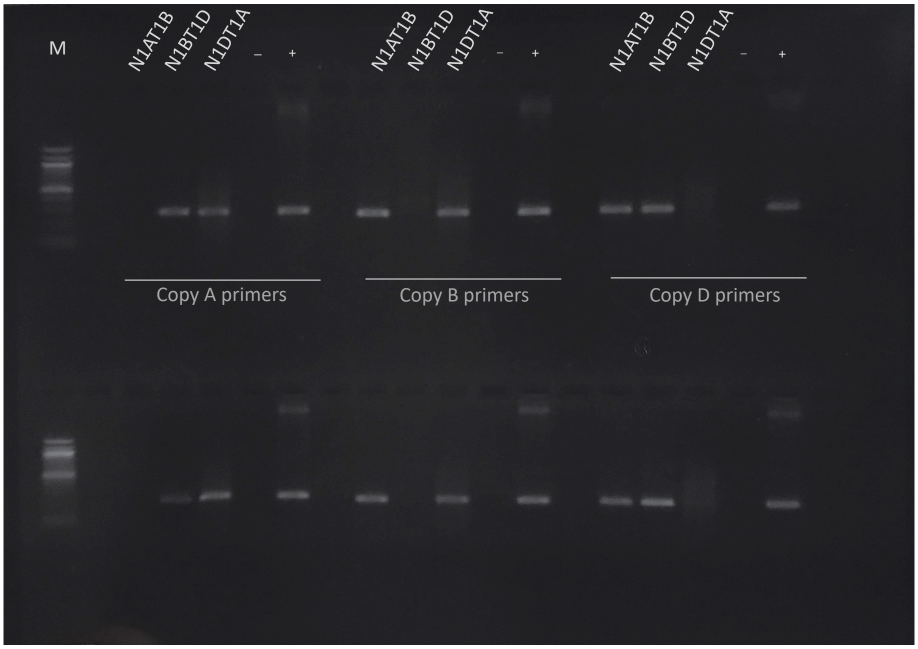

Supplement 10 Differential expression analysis of *TaCLPB* for the wheat developmental time course dataset (ENA: ERP004714) using EdgeR.

TaCLPB	**Grain (2DAA-14DAA)**						**Grain (2DAA-30DAA)**						**Grain (14DAA-30DAA)**					
	**logFC**	**logCPM**	**F**	**PValue**	**FDR**	**Sign**	**logFC**	**logCPM**	**F**	**PValue**	**FDR**	**Sign**	**logFC**	**logCPM**	**F**	**PValue**	**FDR**	**Sign**
TaCLPB-1A	2.6899898	6.0530014	4.4316698	0.0481339	0.2527935	.	0.0664695	6.0530014	0.0030664	0.9563894	1		-2.6235203	6.0530014	4.237639	0.0528178	0.2432796	
TaCLPB-1B	2.3196861	6.4614056	5.9224214	0.0244637	0.1520324	.	-0.5432457	6.4614056	0.3555124	0.5577057	1		-2.8629318	6.4614056	8.6359332	0.0081268	0.0529757	*
TaCLPB-1D	2.3399465	6.3898404	4.4398527	0.0479471	0.2520793	.	-0.2362026	6.3898404	0.0498323	0.8256222	1		-2.5761491	6.3898404	5.2827961	0.0324658	0.1659351	.
TaCLPB-3A	1.4674656	4.4521471	7.8311718	0.0111037	0.0823995	.	-1.0791646	4.4521471	4.3032545	0.0511779	0.2021205		-2.5466302	4.4521471	21.890877	0.0001448	0.0016961	**
TaCLPB-3B	2.891013	4.2443777	12.038398	0.0024224	0.0244469	*	-0.4728292	4.2443777	0.3680307	0.5509125	1		-3.3638423	4.2443777	15.619058	0.0007883	0.0072799	**
TaCLPB-3D	1.7641194	3.7571271	15.638066	0.0007839	0.0099105	**	-1.5788633	3.7571271	12.606911	0.0020083	0.0130135	*	-3.3429827	3.7571271	49.506226	8.03E-07	2.02E-05	***
TaCLPB-4B1	-3.1453215	1.91319	2.8495317	0.2774803	0.8158948	.	-3.1453215	1.91319	4.9343603	0.1483467	0.459172		0	1.91319	0	1	1	
TaCLPB-5A	0.5168521	5.5225679	10.024326	0.0048622	0.0427221	*	-2.1117291	5.5225679	154.85823	7.24E-11	6.03E-09	***	-2.6285812	5.5225679	230.67568	1.95E-12	7.39E-10	***
TaCLPB-4B	-0.1630762	5.4779054	1.7186096	0.2047427	0.6891019		-2.5600458	5.4779054	377.09219	1.94E-14	1.50E-11	***	-2.3969696	5.4779054	334.46735	6.05E-14	6.07E-11	***
TaCLPB-4D	0.7243869	5.5353191	22.360511	0.0001289	0.0023056	**	-2.1216345	5.5353191	178.23914	2.05E-11	2.28E-09	***	-2.8460214	5.5353191	303.14749	1.53E-13	1.18E-10	***
TaCLPB-6A	1.3314943	4.3129848	46.627944	1.24E-06	5.68E-05	**	-0.3216212	4.3129848	2.8068155	0.1094475	0.3661167		-1.6531154	4.3129848	70.58174	5.48E-08	2.14E-06	***
TaCLPB-6B	1.7602412	4.2642228	81.834608	1.68E-08	2.17E-06	**	-0.2055869	4.2642228	1.1805528	0.2901805	0.7372813		-1.9658281	4.2642228	100.65536	3.03E-09	2.01E-07	***
TaCLPB-6D	1.2953668	4.4906376	64.731695	1.08E-07	8.81E-06	**	-0.4189333	4.4906376	6.9627639	0.015758	0.076462	.	-1.7143001	4.4906376	110.72734	1.35E-09	1.05E-07	***

TaCLPB	**Spike (two nodes - Meiosis)**						**Spike (Two nodes - Anthesis)**						**Spike (Meiosis -Anthesis)**					
	**logFC**	**logCPM**	**F**	**PValue**	**FDR**	**Sign**	**logFC**	**logCPM**	**F**	**PValue**	**FDR**	**Sign**	**logFC**	**logCPM**	**F**	**PValue**	**FDR**	**Sign**
TaCLPB-1A	0.0268699	6.0530014	0.0004343	0.983581	1		5.5584721	6.0530014	14.111371	0.0012444	0.0153073	*	5.5316022	6.0530014	13.997928	0.001289	0.0223792	*
TaCLPB-1B	0.130376	6.4614056	0.0186093	0.892858	1		5.4434092	6.4614056	24.062534	8.57E-05	0.0017605	**	5.3130333	6.4614056	23.209002	0.000105	0.0032371	**
TaCLPB-1D	-0.5204398	6.3898404	0.2227802	0.6420445	1		4.8633097	6.3898404	15.073059	0.0009277	0.0120619	**	5.3837495	6.3898404	17.48812	0.0004608	0.0101949	**
TaCLPB-3A	-2.7266665	4.4521471	23.615784	9.53E-05	0.0039109	**	1.3888936	4.4521471	6.9805569	0.015643	0.1154065	.	4.1155602	4.4521471	48.269391	9.64E-07	9.67E-05	**
TaCLPB-3B	-2.4999008	4.2443777	8.9487179	0.0072198	0.0956049	**	0.9153376	4.2443777	1.3560421	0.2579431	0.7943219		3.4152384	4.2443777	15.596042	0.0007937	0.0155463	**
TaCLPB-3D	-2.4678464	3.7571271	28.237294	3.37E-05	0.001799	**	0.4256757	3.7571271	0.9530112	0.3406283	0.930383		2.893522	3.7571271	37.647324	5.41E-06	0.0003437	**
TaCLPB-4B1	0.0620394	1.91319	0.006388	0.9473007	1		3.3874051	1.91319	29.156274	0.0001612	0.00293	**	3.3253657	1.91319	27.959742	0.0001934	0.0051954	**
TaCLPB-5A	0.6216702	5.5225679	14.445834	0.0011224	0.0245152	*	0.891282	5.5225679	29.467603	2.60E-05	0.0006774	**	0.2696118	5.5225679	2.73616	0.1137284	0.5443533	
TaCLPB-4B	0.3750457	5.4779054	9.0657804	0.0069097	0.0926262	*	0.848811	5.4779054	45.932435	1.37E-06	6.77E-05	**	0.4737653	5.4779054	14.452203	0.0011202	0.0200829	*
TaCLPB-4D	0.4071095	5.5353191	7.0932713	0.0149364	0.1599897	.	0.8073365	5.5353191	27.647837	3.82E-05	0.0009241	**	0.4002271	5.5353191	6.8630407	0.01642	0.1482401	.
TaCLPB-6A	-0.1861697	4.3129848	0.9378865	0.3444016	1		0.7319841	4.3129848	14.386203	0.0011431	0.0142809	*	0.9181538	4.3129848	22.488266	0.000125	0.0037055	**
TaCLPB-6B	-0.2665415	4.2642228	1.9656984	0.1762528	0.7622901		0.8794929	4.2642228	21.198283	0.0001721	0.0030906	**	1.1460344	4.2642228	35.584887	7.85E-06	0.0004554	**
TaCLPB-6D	-0.3980276	4.4906376	6.2722834	0.0210435	0.202385	.	0.4188432	4.4906376	6.9531421	0.0158205	0.11638	.	0.8168708	4.4906376	26.174131	5.28E-05	0.001921	**

TaCLPB	**Leaf (first leaf - three tillers)**						**Leaf (first leaf - 2DAA)**						**Leaf (three tillers - 2DAA)**					
	**logFC**	**logCPM**	**F**	**PValue**	**FDR**	**Sign**	**logFC**	**logCPM**	**F**	**PValue**	**FDR**	**Sign**	**logFC**	**logCPM**	**F**	**PValue**	**FDR**	**Sign**
TaCLPB-1A	-0.2037087	6.0530014	0.0283309	0.8680257	1		4.2452175	6.0530014	9.5547566	0.0057657	0.0372704	*	4.4489262	6.0530014	10.296523	0.0044112	0.0426318	*
TaCLPB-1B	-0.1295857	6.4614056	0.0201988	0.8884067	1		3.6442088	6.4614056	13.028049	0.0017521	0.0134236	*	3.7737945	6.4614056	13.80859	0.0013675	0.0163526	*
TaCLPB-1D	-0.3661712	6.3898404	0.1185656	0.7341937	1		3.4091303	6.3898404	8.5921216	0.0082637	0.0505516	*	3.7753015	6.3898404	10.189322	0.004583	0.0439468	*
TaCLPB-3A	2.3264205	4.4521471	18.439296	0.0003545	0.0101318	**	4.7028202	4.4521471	61.258458	1.64E-07	4.48E-06	***	2.3763997	4.4521471	19.351084	0.0002775	0.0043344	**
TaCLPB-3B	0.1114754	4.2443777	0.0201759	0.8884696	1		4.0152835	4.2443777	20.858892	0.0001876	0.0019309	**	3.9038081	4.2443777	19.956733	0.0002367	0.0037852	**
TaCLPB-3D	-0.2765565	3.7571271	0.395699	0.5364464	1		3.2831665	3.7571271	47.743139	1.04E-06	2.15E-05	***	3.5597229	3.7571271	54.769966	3.83E-07	1.70E-05	***
TaCLPB-4B1	9.553481	1.91319	100.08734	3.62E-07	5.96E-05	***	14.2583	1.91319	233.79702	3.20E-09	1.65E-07	***	4.7048192	1.91319	81.439551	1.09E-06	4.08E-05	***
TaCLPB-5A	-0.3644353	5.5225679	4.996733	0.036974	0.3098656	.	-0.1458535	5.5225679	0.8020915	0.3811338	0.9331485		0.2185818	5.5225679	1.8003893	0.1947192	0.7162366	
TaCLPB-4B	0.3337189	5.4779054	7.1925233	0.0143436	0.0804025	.	0.3337189	5.4779054	7.1925233	0.0143436	0.0804025	.	0.722803	5.4779054	33.467331	1.17E-05	0.0003038	***
TaCLPB-4D	-0.2730389	5.5353191	3.2042588	0.0886168	0.5406743	.	0.8534311	5.5353191	30.918056	1.93E-05	0.0002682	***	1.12647	5.5353191	53.312155	4.67E-07	2.01E-05	***
TaCLPB-6A	0.3852977	4.3129848	4.0049554	0.0591379	0.2551865	.	0.3852977	4.3129848	4.0049554	0.0591379	0.2551865	.	1.0206488	4.3129848	27.59508	3.87E-05	0.0008288	***
TaCLPB-6B	-0.1746255	4.2642228	0.8462698	0.3685853	1		0.8778116	4.2642228	21.124729	0.0001754	0.0018236	**	1.0524372	4.2642228	30.182069	2.24E-05	0.0005232	***
TaCLPB-6D	-0.3114279	4.4906376	3.8353992	0.064292	0.4439245		0.2033568	4.4906376	1.6380532	0.2152569	0.659284		0.5147848	4.4906376	10.450197	0.0041773	0.0407655	*

TaCLPB	**Stem (Pseudospike - Two nodes)**						**Stem (Pseudospike - Anthesis)**						**Stem (Two nodes - Anthesis)**					
	**logFC**	**logCPM**	**F**	**PValue**	**FDR**	**Sign**	**logFC**	**logCPM**	**F**	**PValue**	**FDR**	**Sign**	**logFC**	**logCPM**	**F**	**PValue**	**FDR**	**Sign**
TaCLPB-1A	4.8894317	6.0530014	11.436153	0.0029671	0.0494699	*	2.894156	6.0530014	4.6457181	0.0435077	0.2099217	.	-1.9952757	6.0530014	2.519746	0.128131	0.5422641	
TaCLPB-1B	2.6635722	6.4614056	7.4343708	0.0130063	0.1444374	.	1.3674527	6.4614056	2.1073665	0.1621159	0.5599689		-1.2961195	6.4614056	1.9379179	0.1791999	0.670116	
TaCLPB-1D	1.6417337	6.3898404	2.1524396	0.1579109	0.5497706		1.6417337	6.3898404	2.1524396	0.1579109	0.5497706		-1.964991	6.3898404	3.1450958	0.0914022	0.4322078	.
TaCLPB-3A	0.3775378	4.4521471	0.5033693	0.4862225	1		0.0505696	4.4521471	0.0088807	0.9258592	1		-0.3269682	4.4521471	0.373064	0.5482259	1	
TaCLPB-3B	-0.2805604	4.2443777	0.1248179	0.7275676	1		-3.6520509	4.2443777	15.00708	0.0009463	0.0087381	**	-3.3714905	4.2443777	12.922656	0.0018126	0.0206334	*
TaCLPB-3D	-0.5311928	3.7571271	1.4366598	0.2447067	0.9030239		-6.2707017	3.7571271	94.161762	5.29E-09	3.66E-07	***	-5.739509	3.7571271	79.512396	2.12E-08	1.88E-06	***
TaCLPB-4B1	0	1.91319	0	1	1		8.609023	1.91319	96.237859	4.47E-07	1.33E-05	***	8.609023	1.91319	93.944548	5.09E-07	2.34E-05	***
TaCLPB-5A	-0.2493177	5.5225679	2.3378654	0.1419431	0.6789986		0.3282668	5.5225679	4.050345	0.0578398	0.2625577	.	0.5775845	5.5225679	12.478644	0.0020944	0.0232377	*
TaCLPB-4B	0.1473563	5.4779054	1.4023387	0.2502313	0.9098583		0.4022684	5.4779054	10.420839	0.0042209	0.0309973	*	0.2549122	5.4779054	4.1927395	0.0539746	0.2980317	.
TaCLPB-4D	0.0782635	5.5353191	0.2629897	0.6136978	1		0.6450213	5.5353191	17.726019	0.0004312	0.0044775	**	0.5667579	5.5353191	13.709676	0.0014107	0.0167969	*
TaCLPB-6A	-0.0500503	4.3129848	0.0677845	0.797255	1		-1.2796348	4.3129848	42.642459	2.32E-06	5.28E-05	***	-1.2295845	4.3129848	39.446924	3.95E-06	0.0001262	***
TaCLPB-6B	-0.1188883	4.2642228	0.392346	0.5381611	1		-1.4573779	4.2642228	56.092501	3.20E-07	1.00E-05	***	-1.3384896	4.2642228	47.557704	1.07E-06	4.30E-05	***
TaCLPB-6D	-0.4978299	4.4906376	9.7826873	0.0053058	0.0753146	*	-1.7353586	4.4906376	112.22717	1.20E-09	1.15E-07	***	-1.2375287	4.4906376	58.500475	2.33E-07	1.24E-05	***

TaCLPB	**Root (First leaf - three leaves)**						**Root (First leaf - Meiosis)**						**Root (Three leaves - Meiosis)**					
	**logFC**	**logCPM**	**F**	**PValue**	**FDR**	**Sign**	**logFC**	**logCPM**	**F**	**PValue**	**FDR**	**Sign**	**logFC**	**logCPM**	**F**	**PValue**	**FDR**	**Sign**
TaCLPB-1A	3.3638981	6.0530014	6.3177599	0.0206399	0.2429211	.	2.5558202	6.0530014	3.8620319	0.0634492	0.5721175	.	-0.8080779	6.0530014	0.4426093	0.5134742	1	
TaCLPB-1B	1.7217654	6.4614056	3.3670655	0.081448	0.5056407	.	1.0917195	6.4614056	1.3933646	0.2517025	1		-0.6300459	6.4614056	0.474822	0.4987031	1	
TaCLPB-1D	2.206497	6.3898404	3.9000453	0.0622683	0.4412432	.	1.549287	6.3898404	1.9907434	0.1736469	0.8916105		-0.6572099	6.3898404	0.3788797	0.5451528	1	
TaCLPB-3A	-0.2869176	4.4521471	0.2944793	0.5933674	1		-0.6707762	4.4521471	1.5690664	0.2248083	0.9855594		-0.3838586	4.4521471	0.5195896	0.4793679	1	
TaCLPB-3B	0.929245	4.2443777	1.3492567	0.2590993	0.9094364		0.7871202	4.2443777	0.9632579	0.338105	1		-0.1421248	4.2443777	0.0325464	0.8586513	1	
TaCLPB-3D	0.1811153	3.7571271	0.1657843	0.6882158	1		-0.4174622	3.7571271	0.8634141	0.3638777	1		-0.5985774	3.7571271	1.7940415	0.195475	1	
TaCLPB-4B1	0	1.91319	0	1	1		0	1.91319	0	1	1		0	1.91319	0	1	1	
TaCLPB-5A	-0.5213245	5.5225679	10.157918	0.0046348	0.105341	*	-0.2553692	5.5225679	2.4483534	0.1333518	0.8001399		0.2659553	5.5225679	2.654314	0.1189373	1	
TaCLPB-4B	-0.4962178	5.4779054	15.772587	0.0007534	0.0366964	**	-0.4000327	5.4779054	10.272182	0.0044496	0.1387878	*	0.0961851	5.4779054	0.5953256	0.4493983	1	
TaCLPB-4D	-0.5086552	5.5353191	11.029326	0.0034116	0.0874347	*	-0.490093	5.5353191	10.243184	0.0044958	0.1395855	*	0.0185622	5.5353191	0.0147564	0.9045278	1	
TaCLPB-6A	-1.3910703	4.3129848	50.407618	7.05E-07	0.000471	***	-1.3525326	4.3129848	47.784894	1.04E-06	0.0007519	***	0.0385377	4.3129848	0.0399973	0.8435077	1	
TaCLPB-6B	-1.3059982	4.2642228	45.807374	1.40E-06	0.0007558	***	-1.302726	4.2642228	45.619879	1.44E-06	0.0009138	***	0.0032722	4.2642228	0.000296	0.9864443	1	
TaCLPB-6D	-1.0359624	4.4906376	41.7023	2.70E-06	0.001142	***	-0.99075	4.4906376	38.223452	4.89E-06	0.0020059	***	0.0452124	4.4906376	0.0809741	0.7789104	1	

*Significance codes: 0 '***'; 0.001 '**'; 0.01 '*'; 0.05 '.' ;0.1' '; 1*

Supplement 11 Differential expression analysis of *TaCLPB* for the drought and heat dataset (SRA: SRP045409) using EdgeR

	drought (0h - 1h)						drought (0h - 6h)						drought (1h - 6h)					
TaCLPB	logFC	logCPM	F	PValue	FDR	Sign	logFC	logCPM	F	PValue	FDR	Sign	logFC	logCPM	F	PValue	FDR	Sign
TaCLPB-1A	1.22742219	9.94480786	78.4768732	3.56E-07	4.52E-05	***	1.55177503	9.94480786	124.13923	2.00E-08	1.20E-06	***	0.32435284	9.94480786	5.88387042	0.02910728	0.22149913	.
TaCLPB-1B	1.8993758	10.8043076	216.617451	5.10E-10	2.11E-07	***	1.63949025	10.8043076	163.378516	3.34E-09	2.71E-07	***	-0.2598855	10.8043076	4.36057948	0.05518126	0.36015307	
TaCLPB-1D	1.08538429	10.842521	93.0167728	1.25E-07	1.91E-05	***	1.36431924	10.842521	145.168114	7.25E-09	5.14E-07	***	0.27893496	10.842521	6.31530468	0.024574	0.19405505	.
TaCLPB-3A	-0.7730188	10.1644707	51.4796821	4.28E-06	0.0003623	***	-2.5104579	10.1644707	450.615265	3.43E-12	1.03E-09	***	-1.7374391	10.1644707	218.626154	4.80E-10	7.61E-08	***
TaCLPB-3B	1.81735959	9.58279995	28.3295343	0.00010122	0.00511023	**	0.89363907	9.58279995	5.66696397	0.03813482	0.25217145	*	-0.9237205	9.58279995	7.89953051	0.01369198	0.12286823	
TaCLPB-3D	1.71778634	9.3530524	18.8049947	0.01066531	0.20412005	.	1.6609662	9.3530524	16.1604855	0.01799898	0.14119328	.	-0.0568201	9.3530524	0.02664248	0.96902041	1	
TaCLPB-4B1	-0.0760388	2.58831785	0.18343009	0.67484095	1		-3.4039879	2.58831785	291.199479	6.89E-11	1.14E-08	***	-3.3279491	2.58831785	279.767687	9.05E-11	2.11E-08	***
TaCLPB-5A	-0.0830921	9.73358204	1.24147478	0.28361691	1		-0.1832191	9.73358204	6.01963828	0.02758308	0.19682135	.	-0.100127	9.73358204	1.79709405	0.20103345	0.84984392	
TaCLPB-4B	0.17410677	9.81150163	7.5545408	0.01548151	0.26578387	.	0.25257856	9.81150163	15.869821	0.001314	0.01624079	*	0.07847179	9.81150163	1.53364373	0.23556866	0.92853192	
TaCLPB-4D	-0.0969169	9.93081433	1.60455236	0.22556092	1		-0.1886577	9.93081433	6.06005182	0.02714747	0.19438619	.	-0.0917407	9.93081433	1.43214796	0.25093406	0.96121149	
TaCLPB-6A	0.11531509	7.22820245	1.25034553	0.2819727	1		0.19917081	7.22820245	3.70160149	0.0745559	0.41481111	.	0.08385571	7.22820245	0.65622243	0.54524399	1	
TaCLPB-6B	0.01436286	7.95684765	0.01017651	0.92105158	1		-0.1590088	7.95684765	1.21591584	0.28842812	0.99314767		-0.1733717	7.95684765	1.43960925	0.24976013	0.95856609	
TaCLPB-6D	-0.1965707	7.32191553	2.41608965	0.14200556	0.98158241		-0.2155814	7.32191553	2.87774888	0.11152558	0.5489266		-0.0190107	7.32191553	0.0222632	0.88347895	1	

	heat (0h - 1h)						heat (0h - 6h)						heat (1h - 6h)					
TaCLPB	logFC	logCPM	F	PValue	FDR	Sign	logFC	logCPM	F	PValue	FDR	Sign	logFC	logCPM	F	PValue	FDR	Sign
TaCLPB-1A	12.1564299	9.94480786	3621.40866	1.45E-18	7.34E-15	***	7.95723157	9.94480786	2103.5448	6.84E-17	3.01E-13	***	-4.1991983	9.94480786	852.644529	4.01E-14	6.11E-11	***
TaCLPB-1B	10.3684502	10.8043076	3161.63454	3.80E-18	1.32E-14	***	7.12593798	10.8043076	1939.58961	1.22E-16	4.03E-13	***	-3.2425122	10.8043076	580.849026	5.87E-13	3.94E-10	***
TaCLPB-1D	10.1527942	10.842521	3887.09245	8.77E-19	5.46E-15	***	6.77067969	10.842521	2281.69825	3.85E-17	2.03E-13	***	-3.3821145	10.842521	787.087876	7.02E-14	9.12E-11	***
TaCLPB-3A	10.258017	10.1644707	4445.30743	3.38E-19	2.97E-15	***	5.44706963	10.1644707	1879.72403	1.52E-16	4.68E-13	***	-4.8109473	10.1644707	1573.9123	5.33E-16	3.70E-12	***
TaCLPB-3B	14.5259007	9.58279995	1117.18206	5.99E-15	3.50E-12	***	8.91948665	9.58279995	585.146936	5.58E-13	1.91E-10	***	-5.606414	9.58279995	348.395794	2.02E-11	5.12E-09	***
TaCLPB-3D	15.9121621	9.3530524	1756.76747	2.45E-16	2.82E-13	***	10.335509	9.3530524	958.642353	1.76E-14	1.44E-11	***	-5.5766531	9.3530524	521.06726	1.25E-12	6.65E-10	***
TaCLPB-4B1	-0.8209492	2.58831785	20.6745124	0.00043632	0.00518031	**	-3.7541275	2.58831785	346.525634	2.10E-11	3.11E-09	***	-2.9331783	2.58831785	221.915173	4.34E-10	5.34E-08	***
TaCLPB-5A	6.63412915	9.73358204	4884.52188	1.73E-19	2.08E-15	***	5.33921779	9.73358204	3570.01073	1.61E-18	3.00E-14	***	-1.2949114	9.73358204	295.052152	6.30E-11	1.22E-08	***
TaCLPB-4B	6.99897456	9.81150163	7295.20914	9.97E-21	4.97E-16	***	5.18433584	9.81150163	4736.69545	2.15E-19	9.19E-15	***	-1.8146387	9.81150163	780.57611	7.44E-14	9.42E-11	***
TaCLPB-4D	7.33679811	9.93081433	5343.40228	9.14E-20	1.39E-15	***	5.29241649	9.93081433	3355.3377	2.49E-18	4.23E-14	***	-2.0443816	9.93081433	670.290886	2.16E-13	1.96E-10	***
TaCLPB-6A	7.25693247	7.22820245	3034.08257	5.10E-18	1.62E-14	***	4.70226887	7.22820245	1606.45609	4.61E-16	1.02E-12	***	-2.5546636	7.22820245	584.371402	5.63E-13	3.82E-10	***
TaCLPB-6B	8.70441984	7.95684765	2097.59797	6.98E-17	1.10E-13	***	5.47131223	7.95684765	1095.53153	6.87E-15	7.31E-12	***	-3.2331076	7.95684765	481.393441	2.17E-12	9.78E-10	***
TaCLPB-6D	7.02057531	7.32191553	1914.7694	1.33E-16	1.81E-13	***	4.45584487	7.32191553	976.128367	1.55E-14	1.32E-11	***	-2.5647304	7.32191553	387.923129	9.64E-12	2.97E-09	***

	heat+drought (0h - 1h)						heat+drought (0h-6h)						heat+drought (1h-6h)					
TaCLPB	logFC	logCPM	F	PValue	FDR	Sign	logFC	logCPM	F	PValue	FDR	Sign	logFC	logCPM	F	PValue	FDR	Sign
TaCLPB-1A	12.1259616	9.94480786	3610.35964	1.48E-18	7.08E-15	***	6.7481146	9.94480786	1671.42807	3.48E-16	8.82E-13	***	-5.3778469	9.94480786	1248.86225	2.73E-15	6.64E-12	***
TaCLPB-1B	10.4018575	10.8043076	3174.27649	3.70E-18	1.20E-14	***	5.78714641	10.8043076	1444.17896	9.79E-16	1.74E-12	***	-4.6147111	10.8043076	1034.75373	1.03E-14	1.72E-11	***
TaCLPB-1D	10.1722865	10.842521	3896.39106	8.62E-19	5.26E-15	***	5.41107799	10.842521	1651.51978	3.79E-16	9.36E-13	***	-4.7612085	10.842521	1369.80203	1.42E-15	4.25E-12	***
TaCLPB-3A	10.0000935	10.1644707	4306.1051	4.24E-19	3.20E-15	***	4.5185504	10.1644707	1409.89624	1.16E-15	1.96E-12	***	-5.4815431	10.1644707	1915.64921	1.33E-16	8.26E-13	***
TaCLPB-3B	14.2589696	9.58279995	1091.79424	7.04E-15	3.33E-12	***	7.83479485	9.58279995	483.189004	2.11E-12	4.77E-10	***	-6.4241748	9.58279995	423.376406	5.27E-12	1.59E-09	***
TaCLPB-3D	15.876852	9.3530524	1751.70095	2.50E-16	2.56E-13	***	9.21153491	9.3530524	798.966261	6.32E-14	3.35E-11	***	-6.6653171	9.3530524	671.865666	2.13E-13	1.51E-10	***
TaCLPB-4B1	-2.263761	2.58831785	139.559309	9.37E-09	4.10E-07	***	-3.7196825	2.58831785	335.983966	2.59E-11	3.56E-09	***	-1.4559215	2.58831785	54.9036135	2.96E-06	7.32E-05	***
TaCLPB-5A	7.1816438	9.73358204	5449.32237	7.95E-20	1.19E-15	***	5.00654604	9.73358204	3239.92748	3.20E-18	5.49E-14	***	-2.1750978	9.73358204	788.975929	6.90E-14	6.90E-11	***
TaCLPB-4B	7.45927576	9.81150163	7956.03959	5.38E-21	2.68E-16	***	5.2123938	9.81150163	4775.23152	2.03E-19	1.21E-14	***	-2.246882	9.81150163	1161.67582	4.55E-15	9.24E-12	***
TaCLPB-4D	7.81106689	9.93081433	5812.46749	5.02E-20	9.45E-16	***	5.10925332	9.93081433	3181.94653	3.63E-18	5.49E-14	***	-2.7018136	9.93081433	1113.63798	6.12E-15	1.16E-11	***
TaCLPB-6A	7.53570384	7.22820245	3193.86321	3.54E-18	1.19E-14	***	4.35712385	7.22820245	1423.15845	1.09E-15	1.88E-12	***	-3.17858	7.22820245	857.984541	3.83E-14	4.53E-11	***
TaCLPB-6B	8.96211177	7.95684765	2178.64387	5.34E-17	8.18E-14	***	4.86679514	7.95684765	915.415445	2.43E-14	1.72E-11	***	-4.0953166	7.95684765	712.997061	1.40E-13	1.11E-10	***
TaCLPB-6D	7.17104158	7.32191553	1971.43564	1.08E-16	1.35E-13	***	4.12973393	7.32191553	863.711127	3.66E-14	2.27E-11	***	-3.0413076	7.32191553	523.994884	1.20E-12	5.20E-10	***

	heat vs heat+drought (1h)						heat vs heat+drought (6h)					
TaCLPB	logFC	logCPM	F	PValue	FDR	Sign	logFC	logCPM	F	PValue	FDR	Sign
TaCLPB-1A	-0.0304683	9.94480786	0.05831914	0.81261103	1		-1.209117	9.94480786	89.1187515	1.63E-07	2.62E-05	***
TaCLPB-1B	0.03340737	10.8043076	0.07331035	0.79045663	1		-1.3387916	10.8043076	113.681656	3.52E-08	7.87E-06	***
TaCLPB-1D	0.01949225	10.842521	0.03147694	0.86167626	1		-1.3596017	10.842521	147.705812	6.47E-09	2.15E-06	***
TaCLPB-3A	-0.2579235	10.1644707	6.22343785	0.02546608	0.27757577	.	-0.9285192	10.1644707	79.2507493	3.35E-07	4.60E-05	***
TaCLPB-3B	-0.2669311	9.58279995	1.17248932	0.29686272	1		-1.0846918	9.58279995	18.8449724	0.00065057	0.01887116	***
TaCLPB-3D	-0.0353101	9.3530524	0.03097295	0.86277585	1		-1.1239741	9.3530524	30.4109567	7.13E-05	0.00328337	***
TaCLPB-4B1	-1.4428118	2.58831785	57.9115715	2.18E-06	0.00027634	***	0.03444498	2.58831785	0.03165766	0.86128418	1	
TaCLPB-5A	0.54751465	9.73358204	54.1608551	3.20E-06	0.00036464	***	-0.3326717	9.73358204	20.0683846	0.00049681	0.0152962	**
TaCLPB-4B	0.4603012	9.81150163	53.1898314	3.55E-06	0.00039317	***	0.02805796	9.81150163	0.1984351	0.66267572	1	
TaCLPB-4D	0.47426878	9.93081433	38.7803851	2.03E-05	0.00144032	***	-0.1831632	9.93081433	5.80494346	0.03003842	0.32576042	.
TaCLPB-6A	0.27877138	7.22820245	7.79520628	0.01420661	0.18359647	.	-0.345145	7.22820245	11.9105732	0.00380367	0.07316752	*
TaCLPB-6B	0.25769193	7.95684765	3.62928	0.07714115	0.57387377	.	-0.6045171	7.95684765	19.796807	0.00052698	0.01601396	**
TaCLPB-6D	0.15046627	7.32191553	1.49846743	0.24074971	1		-0.3261109	7.32191553	7.01225106	0.01887164	0.23663727	.

*Significance codes: 0 '***'; 0.001 '**'; 0.01 '*'; 0.05 '.' ;0.1' '; 1*
